# Alkene‐Linked Covalent Organic Frameworks Boosting Photocatalytic Hydrogen Evolution by Efficient Charge Separation and Transfer in the Presence of Sacrificial Electron Donors

**DOI:** 10.1002/advs.201902988

**Published:** 2020-05-06

**Authors:** Chunshao Mo, Meijia Yang, Fusai Sun, Junhua Jian, Linfeng Zhong, Zhengsong Fang, Jiangshan Feng, Dingshan Yu

**Affiliations:** ^1^ Key Laboratory for Polymeric Composite and Functional Materials of Ministry of Education Key Laboratory of High Performance Polymer‒based Composites of Guangdong Province School of Chemistry Sun Yat‐sen University Guangzhou 510275 China

**Keywords:** alkene linkages, charge separation, charge transfer, covalent organic frameworks, photocatalysis

## Abstract

Covalent organic frameworks (COFs) are potential photocatalysts for artificial photosynthesis but they are much less explored for photocatalytic hydrogen evolution (PHE). COFs, while intriguing due to crystallinity, tunability, and porosity, tend to have low apparent quantum efficiency (AQE) and little is explored on atomistic structure–performance correlation. Here, adopting triphenylbenzene knots and phenyl linkers as a proof of concept, three structurally related COFs with different linkages are constructed to achieve a tunable COF platform and probe the effect of the linkage chemistry on PHE. Cyano‐substituted alkene‐linked COF (COF–alkene) yields a stable 2330 µmol h^−1^ g^−1^ PHE rate, much superior to imine‐ and imide‐linked counterparts (<40 µmol h^−1^ g^−1^) under visible light irradiation. Impressively, COF–alkene achieves an AQE of 6.7% at 420 nm. Combined femtosecond transient absorption spectroscopy and theoretical calculation disclose the critical role of cyano‐substituted alkene linkages toward high efficiency of charge separation and transfer in the presence of sacrificial electron donors—the decisive key to the superior PHE performance. Such alkene linkages can also be extended to design a series of high‐performance polymeric photocatalysts, highlighting a general design idea for efficient PHE.

## Graphical abstract

Cyano‐substituted alkene linkages enable high‐efficiency of charge separation and transfer in covalent organic frameworks in the presence of sacrificial electron donors, which not only results in much superior photocatalytic hydrogen evolution performance to imide‐ and imine‐linked counterparts together with the apparent quantum efficiency of 6.7% at 420 nm, but also renders a general design idea for advanced polymeric photocatalysts.

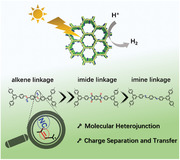

Artificial photosynthesis by converting solar energy into hydrogen energy is an appealing and sustainable strategy to address the global environmental and energy issues.^[^ ^1^ ^]^ Organic semiconductors are regarded as intriguing catalysts for photocatalytic solar‐to‐hydrogen production or water splitting owing to their synthetic tunability and readily tailored optical and electronic properties.^[^ ^2^ ^]^ Carbon nitrides,^[^ ^1^, ^3^ ^]^ conjugated microporous polymers,^[^ ^4^ ^]^ linear conjugated polymers,^[^ ^5^ ^]^ and covalent triazine frameworks^[^ ^6^ ^]^ have been explored as photocatalysts for water splitting. However, they are in general amorphous or semicrystalline, constraining the transport of photogenerated charges to the catalyst surface.^[^ ^7^, ^9^ ^]^ Furthermore, it is still a challenging task to build atomistic structure–performance correlation. Recently, covalent organic frameworks (COFs) have been emerged as promising photoactive materials for artificial photosynthesis due to their multiple merits including long‐range order, broad light harvesting and, sometimes, high‐carrier mobility and good stability.^[^ ^8^ ^]^ In particular, many COFs possess excellent visible light response in a broad region of the solar spectrum (450–700 nm). Despite huge potential, however, much less was exploited for photocatalytic hydrogen evolution (PHE) except for only a few COFs.^[^ ^9^ ^]^ These reported COFs still suffer from unsatisfactory apparent quantum efficiency (AQE) (0.1–4.8% at 420 nm), encumbering the full demonstration of their photocatalytic potential. On the other hand, the existing COFs for PHE are mainly based on imine/enamine, hydrazone, and azine linkages.^[^ ^9^ ^]^ Recently, several groups developed a few sp^2^ carbon‐conjugated COFs via a C–C linkage, which enable a full π conjugation, desirable for optoelectronics and electrochemistry.^[^ ^10^ ^]^ While imine and imide linkages are less efficient in supporting π delocalization due to high polarization of heteroatom N in the skeletons by comparison.^[^ ^8c^ ^]^ Yet, owing to great synthesis difficulty, sp^2^ carbon‐linked COFs were scantly explored for PHE expect for only two examples.^[^ ^9^, ^10^ ^]^

To construct advanced COF‐based photoactive materials for water splitting, previous strategies focus on the molecular design of the knot or linker with a specific linkage. For example, several heteronuclear molecular functionalities such as sulfone‐, triazine‐ or heptazine‐based units or diacetylene moieties were incorporated into the knots or linkers to effectively tune the physicochemical properties of COFs and hence promote PHE.^[^ ^9^ ^]^ To date, very little attention was paid to the role of the linkage chemistry in the rational design of COF‐based photocatalysts, while the linkage tends to determine the chemical stability and electronic communication of COFs, which is of significance for the photochemical process. Furthermore, photoexcited charge transfer in COFs is critically important for photocatalysis but there still lacks a lucid understanding on the dynamic.

Here, using triphenylbenzene knots and phenyl linkers as a proof of concept, we build a tunable COF platform by constructing three structurally related COFs with imine‐, imide‐, and cyano‐substituted alkene‐linkages, respectively, and probe how linkages influence photocatalysis via combined experimental and theoretical studies. These constructed COFs while adopting different linkages, display similar topologies and stacking modes. We observe a PHE activity trend: COF–alkene >> COF–imide > COF–imine. Femtosecond transient absorption (fs‐TA) spectroscopy and theoretical calculation render a lucid understanding on such trend, disclosing the critical role of the linkage in tuning charge separation and transport for efficient photocatalysis. We discover that cyano‐substituted alkene linkages with not only stronger electron‐withdrawing ability but also stronger electron delocalization enable the formation of favorable molecular heterojunctions through the framework to facilitate charge separation and transfer especially in the presence of sacrificial electron donors relative to other two linkages. As such, COF–alkene yields an AQE of 6.7% at 420 nm, together with a notably higher H_2_ evolution rate of 2330 µmol h^−1^ g^−1^ relative to the imine‐ and imide‐linked analogues (<40 µmol h^−1^ g^−1^). Noteworthy, we adopt identical alkene linkage to design other two new conjugated polymers, demonstrating high PHE activities. This highlights a general design idea for advanced polymeric photocatalysts.

In our experiments, all three COFs were synthesized similar to literature‐reported methods but with some modification, particularly for COF–alkene, different solvents and catalysts from the literature were adopted in our case, which results in distinct morphologies and physicochemical properties (see more details in Tables S1–S3, Supporting Information and **Figure** **1**a–d).^[^ ^10^, ^11^ ^]^ We applied 1,3,5‐tris‐(4‐formylphenyl)benzene (Figure S1, Supporting Information) and p‐phenylenediacetonitrile to produce COF–alkene via a Knoevenagel condensation reaction.^[^ ^10g^ ^]^ We used 1,3,5‒tris(4‒aminophenyl)benzene (TAPB) and pyromellitic dianhydride to construct COF–imide.^[^ ^11a^ ^]^ We also performed a Schiff‐base condensation reaction between TAPB and terephthaladehyde (TPAL) for the formation of COF–imine.^[^ ^11b^ ^]^ As indicated by powder X‐ray diffraction (PXRD) patterns in Figure 1e–g and Figures S2–S4 (Supporting Information), all three linkages give rise to crystalline COFs with AA stacking modes. The effective linkage formation in three COFs was verified by Fourier transform infrared (FT‐IR) and solid‐state ^13^C nuclear magnetic resonance (NMR) spectroscopty. As revealed by FT‐IR spectra, the characteristic absorption peak at 2213 cm^−1^ is ascribed to the cyano side group (C=C–CN) for COF–alkene and the observed peaks at 1776, 1723, and 1365 cm^−1^ in COF–imide prove the existence of the five‐membered imide rings, while the peak at 1620 cm^−1^ is attributed to the stretching vibration of C=N for COF–imine (Figures S5–S7, Supporting Information). In the ^13^C NMR spectroscopy, the chemical shift of 110.7 ppm in COF–alkene, 162.9 ppm in COF–imide, and 157.7 ppm in COF–imine can be attributed to the formation of C=C bonds, imide linkages, and C=N bonds, respectively (Figures S8–S10, Supporting Information). Thermogravimetric analysis indicates that all three COFs have excellent thermal stability (>400 °C) (Figures S11–S13, Supporting Information). Scanning electron microscopy (SEM) and transmission electron microscopy (TEM) observation in Figure 1h–j and Figures S14–S16 (Supporting Information) reveals that COF–alkene has a well‐defined fiber‐like morpholgy, different from previously reported sp^2^ carbon‐linked 2DPPV with sheet‐like morpholgy,^[^ ^10g^ ^]^ while COF–imine and COF–imide are irregular agglomerates. We tried to observe the lattice structure by TEM, but failed due to the electron beam damage. The porosity of these COFs was evaluated by nitrogen sorption tests at 77.3 K. The calculated Brunauer–Emmett–Teller (BET) surface areas for COF–alkene, COF–imide, and COF–imine were 185, 1664, and 248 m^2^ g^−1^, respectively (Figures S17–S19, Supporting Information). Of note, the surface areas for three COFs are lower than theoritically predicted values with a perfectly crystalline structure (2160, 1824, and 2302 m^2^ g^−1^ for COF–alkene, COF–imide, and COF–imine, respectively). Such differences are often observed in some previously reported COF materials in the literatures.^[^ ^10d–f^ ^]^ Especially for many previously reported sp^2^ carbon‐linked COFs, the BET surface areas tend to be lower than the theoretically predicted values (2DPPV (472 m^2^ g^−1^),^[^ ^10g^ ^]^ TP‐COF (232 m^2^ g^−1^),^[^ ^10e^ ^]^ 2D CCP‐HATN (317 m^2^ g^−1^),^[^ ^10b^ ^]^ sp^2^c‐COF‐2 (322 m^2^ g^−1^),^[^ ^10c^ ^]^ and Bpy‐sp^2^c‐COF (432 m^2^ g^−1^).^[^ ^10d^ ^]^) This could be caused by the intrinsic low reversibility of Knoevenagel condensation reaction accompanied by high‐density stacking faults in the structure according to the literature.^[^ ^10e^ ^]^ Notably, the surface areas for our COF–imide and COF–imine are larger than those of previously reported imide‐linked PI‐COF‐2 (1297 m^2^ g^−1^)^[^ ^11a^ ^]^ and imine‐linked TPB‐TP‐COF (16 m^2^ g^−1^)^[^ ^11b^ ^]^ with similar molecular structures but different synthesis conditions. On the other hand, water wetting generally matters during PHE because it is vital to afford excellent catalyst dispersiblity and favorable interaction with water and the sacrificial donor. Also, the linkages have great influence on the hydrophobicity, the contact angles of three COFs with pure water are in the following order: COF–imide (0°) < COF–alkene (35°) < COF–imine (120°) (Figures S20–S22, Supporting Information).

**Figure 1:**
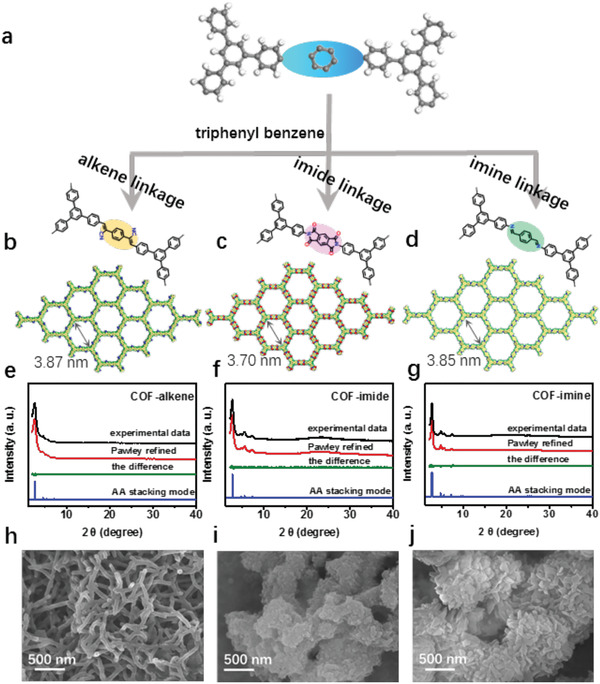
a) Chemical structure of three COFs with different linkages. b–d) Simulated crystal structure of many layers of COF–alkene, COF–imide, and COF–imine, respectively. PXRD patterns of e) COF–alkene, f) COF–imide, and g) COF–imine, SEM images of h) COF–alkene, i) COF–imide, and j) COF–imine, respectively.

The photophysical properties of three COFs were first investigated by UV–vis diffuse reflectance spectra (DRS). As shown in **Figure** **2**a, all three COFs in solid states present similar absorption profiles featuring the wide absorption in the visible light region. The optical bandgaps of COF–alkene, COF–imide, and COF–imine were calculated according to the Tauc plots to be 2.34, 2.00, and 2.46 eV, respectively (Figures S23–S25, Supporting Information), which are all desirable for PHE that demands a minimal bandgap of 1.8 eV.^[^ ^1^, ^9^ ^]^ Mott–Schottky tests^[^ ^12^ ^]^ were performed to determine the LUMO levels of COFs with −0.83, −0.86, and −1.28 eV versus NHE for COF–alkene, COF–imide, and COF–imine, respectively (Figure 2b; Figures S26–S28, Supporting Information), which are all more negative than the thermodynamic proton reduction potential. Clearly, COF–imide can absorb maximum visible light due to the narrowest bandgap while COF–imine has the highest LUMO level, implying the strongest ability to transfer electrons to the Pt reaction center for PHE. Despite different linkages lead to some differences in the optical bandgap and LUMO level of COFs, the above results indicate the theoretical feasibility of all three COFs for PHE.

**Figure 2:**
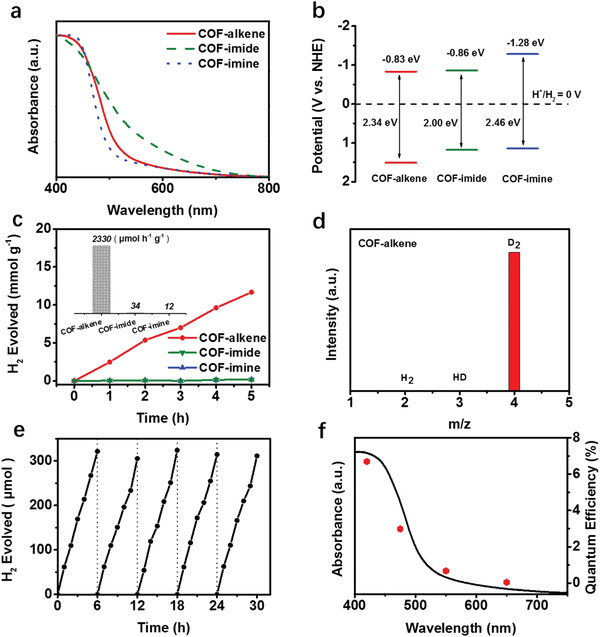
a) UV–vis diffuse reflectance spectra (DRS) of COF–alkene, COF–imide, and COF–imine. b) Energy diagram of three COFs. c) PHE activities of three COF catalysts under visible light irradiation (*λ* > 420 nm) using TEOA as sacrificial agent and Pt as cocatalyst. The inset is the comparison of PHE rate. d) Isotope labeling experiment using D_2_O replace H_2_O for PHE exhibiting the evolution of D_2_ gas, demonstrating the nonoxidative nature of COF material. e) Typical time course of hydrogen production under visible light irradiation using COF–alkene for 30 h. f) Wavelength‐dependent AQE of PHE by COF–alkene photocatalysts. The UV–vis DRS of the photocatalyst is superimposed for comparison.

PHE experiments were performed under visible light irradiation (*λ* > 420 nm) using triethanolamine (TEOA) as a sacrificial electron donor and photodeposited the optimal amount 3% Pt from H_2_PtCl_6_ as a cocatalyst. According to the steady‐state photoluminescence spectroscopy (Figure S29, Supporting Information), COF–alkene/Pt exhibits much lower emission intensity as compared to COF–alkene, indicating much weaker charge recombination rate after Pt deposited on the surface of COF–alkene.^[^ ^3b^ ^]^ Consequently, COF–alkene yields continuous and stable hydrogen production with an average rate of 2330 µmol h^−1^ g^−1^. In sharp contrast, COF–imide and COF–imine, structurally analogous to COF–alkene, present considerably low PHE rates of 34 and 12 µmol h^−1^ g^−1^ (Figure 2c). Even when the PHE activities are normalized to their BET surface areas to exclude the effect of the surface area, COF–alkene still exhibits the best performance (Figure S30, Supporting Information). These results highlight the distinct advantage of alkene linkage in enhancing the PHE activity with respect to other two counterparts. The control experiments within the absence of light or photocatalysts or cocatalyst under otherwise identical conditions do not exhibit detectable amounts of hydrogen. We also conducted isotope labeling experiment to prove that the source of hydrogen indeed comes from water (Figure 2d). Further, a long‐term PHE test reveals that no noticeable activity decay is observed over 30 h for COF–alkene (Figure 2e). After the PHE test, the chemical composition, structure, and crystallinity of COF–alkene were tested by FT‐IR, UV–vis DRS, TEM, nitrogen sorption test, and PXRD characterizations, respectively. It was found that the characteristic XRD peak of the post‐PHE COF–alkene is not as sharp as the initial sample (Figure S31, Supporting Information), which together with the slight decrease of the specific surface area (Figure S32, Supporting Information) indicates the loss of crystallinity similar to the case of some reported COF‐based photocatalysts.^[^ ^9a–c^ ^]^ However, the composition, chemical structure, and optical absorption of COF–alkene remain almost unchanged after the PHE test (Figures S33–S35, Supporting Information). COF–imide and COF–imine display similar photostability within 5 h photocatalytic experiment (Figures S36 and S37, Supporting Information). Furthermore, ex situ SEM imaging was conducted to probe the particle sizes of three COFs under photocatalytic condictions. It was found that three COFs appear in the aggregated form and have similar particle size range of about 2–7 µm (Figure S38, Supporting Information), thus largely excluding the effect of particle sizes on the observed huge PHE performance difference of three COF materials. The AQE was tested under monochromatic incident light of 420, 475, 550, and 650 nm, respectively. Clearly, the AQE increases with the reduced wavelength in accordance with the optical absorption spectrum (Figure 2f). Impressively, the AQE at 420 nm for COF–alkene reaches 6.7%, which is higher than those of many reported photocatalysts based on COFs, although it is still not as good as those of some state‐of‐the‐art conjuagated polymer photocatalysts (Table S4, Supporting Information).

To provide further insights into the role of the linkage chemistry in determining the PHE activities of COFs, the fs‐TA spectroscopy of three COFs were conducted with 0.1 mg mL^−1^ dispersion in aqueous solution.^[^ ^8^, ^13^ ^]^ We first carried out fs‐TA spectroscopy of COF–alkene without TEOA to probe the detailed spectral changes on different scales as shown in Figure S39 of the Supporting Information, the transient absorption evolution from 1.5 to 10 ps displays detailed spectra change occurred on various time scales. Upon excitation at 400 nm, the increase of positive transient absorption at 700 nm in 1.5–1.9 ps for COF–alkene is indicative of the photoexciton generation accompanied by intramolecular charge transfer in the framework. The steady absorption character from 2.2 to 2.4 ps corresponds to the intramolecular charge transfer state of the donor–acceptor (D–A) structure, while the reduced transient absorption in 2.5–10 ps represents the electron–hole recombination process. As for COF–imide and COF–imine, ignorable excited state absorption or only weak absorption on shorter timescales of 1.5–2.1 ps was observed (Figures S40 and S41, Supporting Information), while the negligible absorption reflects a weak absorption corss‐section for trapped electrons.^[^ ^13d^ ^]^ In addition, the fs‐TA spectroscopy of three COFs dispersed in water containing 30% TEOA was carried out to explore the charge carrier dynamics of COFs in the presence of the electron donor of the scavenger. As can be seen from **Figure** **3**, all three COFs present a more positive signal in the 500–760 nm range upon the addition of the electron donor (TEOA), reflecting a significant enhancement of the relative amplitude of the excited state absorption in comparison with the case without TEOA, which can be assigned to higher electron polaron yield, more efficient charge separation and accumulation of long‐lived electrons (Figure 3a).^[^ ^13^ ^]^ Based on the analysis from the kinetics of three COFs (Figure 3b,d,f), similar to the case without TEOA, COF–alkene displays a long‐lived excited state absorption decay of 705 ps, much larger than those of COF–imide (146 ps) and COF–imine (263 ps), benefiting from larger photogenerated electron population for the COF–alkene (electron polarons). In principle, the long lifetime of excited state absorption with TEOA is in line with the high efficiency of charge separation and transfer,^[^ ^13^ ^]^ which is strongly correlated with the linkage species in COFs in our case. Different from other two linkages, the presence of electron‐withdrawing cyano‐substituents in the alkene linkage in combination with electron‐donating triphenylbenzene knots in COF–alkene enables the formation of D–A molecular heterojunction. As demonstrated in the literatures,^[^ ^6^, ^8^ ^]^ due to the strong push–pull interaction between D and A, the photoexcited electrons are prone to transfer from D to A, thereby increasing the spatial electron–hole separation. Consequently, COF–alkene with cyano‐substituted alkene linkages exhibits more efficient charge separation and transfer, thus resulting in the best PHE performance.^[^ ^13^ ^]^

**Figure 3:**
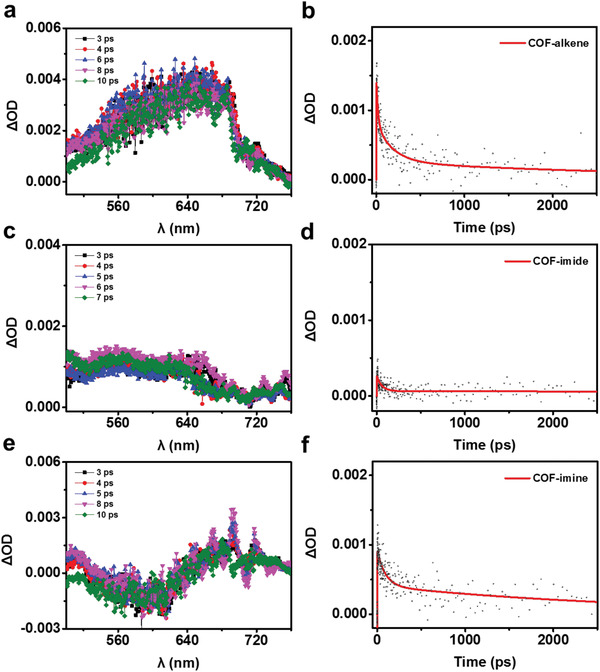
The fs‐TA spectra obtained from suspensions of a) COF–alkene, b) COF–imide, and c) COF–imine in 0.1 mg mL^−1^ aqueous solution containing 30% TEOA, and the corresponding kinetics of characteristic fs‐TA absorption bands observed at 700 nm for the spectra observed of b) COF–alkene, d) COF–imide, and f) COF–imine, respectively.

For three COFs, the identical knot and linker are bridged by different linkages, forming three molecular junctions. To probe the charge transport capability across three linkages, we sandwiched three molecular junctions between two electrodes and calculated the transmission functions by density functional theory calculation (**Figure** **4**a). As observed in Figure 4b, different linkages lead to the marked difference in the transmission gap, determined by the part with the largest HOMO–LUMO gap, which can reflect charge carrier conductance_._ Generally, smaller transmission gap implies facilitated charge transport.^[^ ^14^ ^]^ The P_11_ (HOMO) and P_12_ (LUMO) peaks for alkene linkage center at −1.65 and 0.74 eV, determining the gap of 2.39 eV while the P_21_ and P_22_ peaks at −2.01 and 1.02 eV for imide linkage correspond to the gap of 3.03 eV and the P_31_ and P_32_ peaks at −1.85 and 0.86 eV for imine linkage give the gap of 2.71 eV. Apparently, the alkene linkage affords the smallest gap (COF–alkene < COF–imine < COF–imide), indicating more efficient charge transport over the molecular skeleton with respect to the imine and imide linkages.^[^ ^14^ ^]^

**Figure 4:**
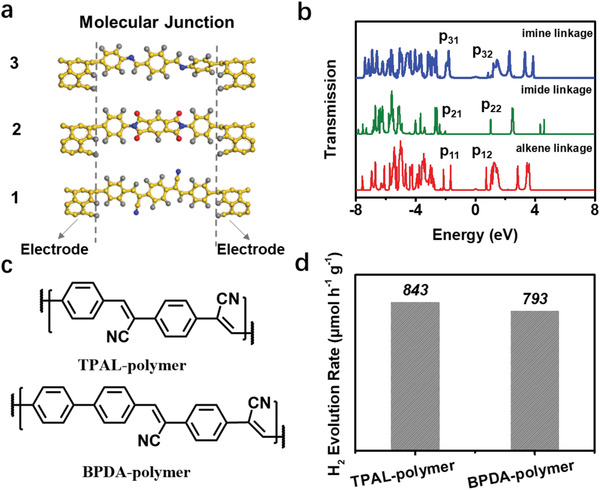
a) Molecular junctions of COF–alkene (1), COF–imide (2), and COF–imine (3) and b) their transmission functions. [P_11_, P_12_], [P_21_, P_22_], and [P_31_, P_32_] present the [HOMO, LUMO] of the transmission functions of COF–alkene, COF–imide, and COF–imine, respectively. c) Chemical structure and d) PHE activities of TPAL–polymer and BPDA–polymer.

Generally, the PHE of COFs is ascribed to a complex interplay of multiple electronic and structural factors. As demonstrated above, we explored the correlation of the optical bandgaps, crystallinity, stacking modes, porosity, and water‐wetting property among three COFs. With the similar stacking mode, particle size, lowest crystallinity and surface area, moderate light harvesting, and moderate water‐wetting property, the COF–alkene achieved the highest PHE activity. As such, we reason that among the above‐mentioned multiple factors the charge separation and transfer efficiency in the presence of TEOA is the central factor determining the PHE event of COFs.^[^ ^12b^ ^]^ Taken together, the superior PHE activity of COF–alkene should benefit from the strong withdrawing ability and electron delocalization of cyano‐substituted alkene linkages, which creates favorable molecular heterojunctions for highly efficient charge separation and transfer especially in the presence of sacrificial electron donors.

To further confirm the critical role of cyano‐substituted alkene linkages in boosting the PHE activity, we synthesized two novel alkene‐linked conjugated polymers based on TPAL and 4,4′‒biphenyldicarboxaldehyde (BPDA) building blocks (denoted as TPAL–polymer and BPDA–polymer, Figure 4c and Figure S42, Supporting Information) following the same protocol as COF–alkene. The UV–vis DRS reveal that both TPAL–polymer and BPDA–polymer have wide visible light absorption and narrow bandgaps of 2.07 and 2.21 eV (Figure S43, Supporting Information). Water contact angle measurements were also conducted to demonstrate the superhydrophilicity of the two polymers (Figures S44 and S45, Supporting Information). Importantly, they yield high PHE rates of 843 and 793 µmol h^−1^ g^−1^ under visible light irradiation (*λ* > 420 nm) (Figure 4d; Figure S46, Supporting Information). To exclude the effect of palladium (Pd) impurity in the materials, we carried out inductively coupled plasma mass spectrometry. As exhibited in Table S5 of the Supporting Information, all of the materials contain <1 ppm Pd impurity, indicating the COF–alkene, TPAL–polymer, and BPDA–polymer indeed are the active composition instead of Pd impurity boosting PHE.^[^ ^13a^ ^]^ This finding indicates the generality of the design concept by engineering the linkage for efficient polymeric photocatalysts.

In summary, we have established a tunable COF platform using triphenylbenzene as knots and phenyl as linkers but with different linkages and elucidated the role of the linkage chemistry on visible light photocatalysis. Systematic property variation of several structurally related COFs indicates that the linkage chemistry can effectively tune the structural and optoelectronic properties of COFs and hence PHE activity. A PHE activity trend COF–alkene >> COF–imide > COF–imine is achieved. COF–alkene yields an AQE of 6.7% at 420 nm together with a drastically higher PHE rate of 2330 µmol h^−1^ g^−1^ than those of imide‐ and imine‐linked counterparts (<40 µmol h^−1^ g^−1^). Noteworthy, although the AQE of COF–alkene is still lower than that of some state‐of‐the‐art conjugated porous polymer PHE catalysts,^[^ ^15^ ^]^ the PHE activity of alkene‐linked COF still holds great room for further improvement by introducing heteronuclear molecular functionalities such as triazine moieties in the linkers.^[^ ^9b,d,f^ ^]^ Combined fs‐TA spectroscopy and theoretical calculation reveal the vital role of cyano‐substituted alkene linkages toward high efficiency of charge separation and transfer especially in the presence of sacrificial electron donors enabled by its strong electron withdrawing ability and electron delocalization, which is the decisive key to superior PHE performance of COF–alkene. Interestingly, alkene linkages can be used to design a series of other conjugated polymers with high PHE activities. Our finding renders some fundamental insight into the correlation between the linkage chemistry and photochemical event. This study highlights a general guideline toward the rational design of advanced polymeric photocatalysts for solar‐to‐fuel conversion.

## Conflict of Interest

The authors declare no conflict of interest.

## Supporting information

Supporting Information
