# Supplementary material for: Alkene‐Linked Covalent Organic Frameworks Boosting Photocatalytic Hydrogen Evolution by Efficient Charge Separation and Transfer in the Presence of Sacrificial Electron Donors
Source: Adv Sci (Weinh). 2020 May 6;7(12):1902988. doi: 10.1002/advs.201902988 (PMC7312270; doi:10.1002/advs.201902988)
Supplement: Supplementary file 1 — Supporting Information [file ADVS-7-1902988-s001.pdf]

Copyright WILEY-VCH Verlag GmbH & Co. KGaA, 69469 Weinheim, Germany, 2020.

Supporting Information

Alkene–Linked Covalent Organic Frameworks Boosting Photocatalytic Hydrogen Evolution by Efficient Charge Separation and Transfer in the Presence of Sacrificial Electron Donors

*Chunshao Mo, Meijia Yang, Fusai Sun, Junhua Jian, Linfeng Zhong, Zhengsong Fang, Jiangshan Feng, and Dingshan Yu**

Chemicals and Materials

All reagents and chemicals used were obtained from commercial sources unless otherwise noted. NaOH was purchased from General-Reagent. P-phenylenediacetonitrile (PDAN), pyromellitic dianhydride (PMDA), 4,4’-biphenyldicarboxaldehyde (BPDA), mesitylene, 1,4-dioxane, N-methylpyrrolidone (NMP), isoquinoline, o-dichlorobenzene (o-DCB), n-butanol, tetrahydrofuran (THF) were purchased from Energy Chemical. Terephthaladehyde (TPAL) and 1,3,5-tris(4-aminophenyl)benzene (TAPB) were purchased from TCI. Nafion solution was purchased from Sigma-Aldrich. 1,3,5-tris(4-formylphenyl)benzene (TFPB) was synthesized according to the literature-reported method.[1]

Characterization

Field-emission scanning electron microscopy (SEM) images were taken on a Hitachi S4800 SEM instrument at an acceleration voltage of 5.0 kV. Transmission electron microscopy (TEM) images were recorded on a JEM-2010 HR field-emission electron microscope at an acceleration voltage of 200 kV. Thermogravimetric analysis (TGA) was conducted on a TG209F1 thermal analyzer at a heating rate of 10 ℃ min^{-1} in N_2. The Fourier transform infrared (FTIR) spectra were obtained on a Thermo Nicolet Nexus 670 spectrometer. Solid-state ^{13}C NMR spectra were obtained on an Agilent DD2 600 MHz NMR spectrometer. Powder X-ray diffraction (PXRD) patterns were collected on a Rigaku Smartlab with Cu Kα source (λ = 1.5418 Å, 40 kV and 30 mA). UV-Vis diffuse reflectance spectra (DRS) were performed on a UV-Vis spectrophotometer (UV-2501PC, Shimadzu) by testing the reflectance of powders in the solid state. Nitrogen sorption isotherms were measured on a Micromeritics ASAP-2460 volumetric gas sorption instrument. The Brunauer-Emmett-Teller (BET) method was carried out to calculate the specific surface area using an ASAP 2460 system.

Materials Synthesis

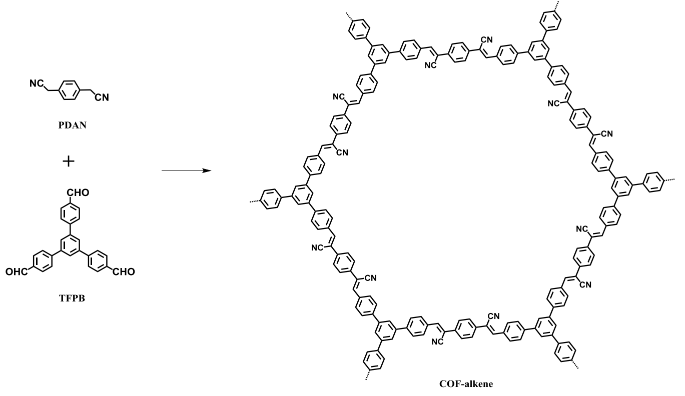

Scheme S1. Synthesis of COF-alkene

Synthesis of COF-alkene. The COF-alkene (also named 2DPPV) in this work was synthesized similar to previously reported method with some modification [2,3] (Table S1). A pyrex tube was charged with 1,3,5-tris(4-formylphenyl)benzene (TFPB, 0.25 mmol), p-phenylenediacetonitrile (PDAN, 0.4 mmol), mesitylene/1,4-dioxane (8 ml, 1/5 by vol.) and aqueous NaOH solution (1.6 mL, 6 M). The mixture was degassed by freeze-pump-thaw technique for three times and sealed under vacuum, and then heated under 90 ℃ for 3 days. After being cooled to room temperature, the precipitate was isolated by filtration and Soxhlet extracted by THF, ethanol and water. The solid was freeze-dried for 24 h to obtain the final product in 86 % isolated yield. The computationally predicted surface area for the atomistic model of a perfectly crystalline structure of COF-alkene is 2160 m^2g^{-1}.

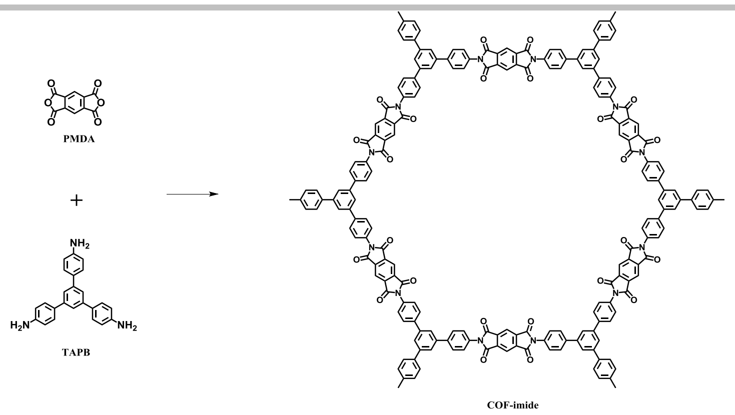

Scheme S2. Synthesis of COF-imide

Synthesis of COF-imide. The COF-imide (also named PI-COF-2) was synthesized according to the reported method[4] (Table S2). A Pyrex tube was charged with 1,3,5-tris(4-aminophenyl)benzene (TAPB, 0.1 mmol), pyromellitic dianhydride (PMDA, 0.15 mmol) in a mixture solution of 2 ml N-methyl pyrrolidone (NMP)/2 mL mesitylene/0.4 ml isoquinoline/0.2 mL water. After being degassed by freeze-pump-thaw technique for three times and then sealed under vacuum, the tube was placed in an oven at 200 ℃ for 5 days. Subsequently, the precipitate was isolated by filtration and Soxhlet extracted by acetone, ethanol and water. The solid was freeze-dried for 24 h to obtain the final product in 85 % isolated yield. The computationally predicted surface area for the atomistic model of a perfectly crystalline structure of COF-imide is 1824 m^2g^{-1}.

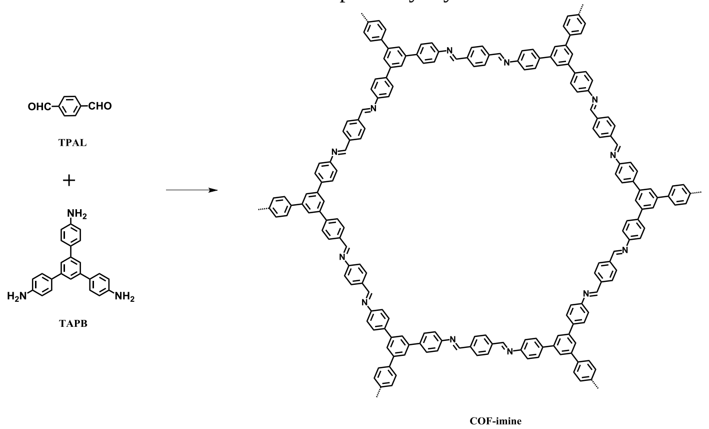

Scheme S3. Synthesis of COF-imine

Synthesis of COF-imine. The COF-imine (also named TPB-TP-COF) was synthesized similar to the literature-reported method with some modification[5] (Table S3). A Pyrex tube was charged with 1,3,5-tris(4-aminophenyl)benzene (TAPB, 0.20 mmol), terephthaladehyde (TPAL, 0.24 mmol) in a solution of 4 mL o-DCB/4 ml n-butanol and 1 mL of 6 M aqueous acetic acid. The tube was sealed under vacuum after being degassed by freeze-pump-thaw technique for three times. The reaction mixture was heated at 120 ℃ for 3 days. Afterwards, the precipitate was isolated by filtration and Soxhlet extracted by THF ethanol and water. The solid was freeze-dried for 24 h to obtain the final product in 81 % isolated yield. The computationally predicted surface area for the atomistic model of a perfectly crystalline structure of COF-imine is 2302 m^2g^{-1}.

Synthesis of TPAL-polymer and BPDA-polymer. A pyrex tube was charged with terephthaladehyde (TPAL, 0.4 mmol) or 4,4'-biphenyldicarboxaldehyde (BPDA, 0.4 mmol), p-phenylenediacetonitrile (PDAN, 0.4 mmol), mesitylene/1,4-dioxane (8 ml, 1/5 by vol.) and aqueous NaOH solution (1.6 ml, 6M). The mixture was degassed by freeze-pump-thaw technique for three times and sealed under vacuum, and then heated under 90 ℃ for 3 days. After cooled to room temperature, the precipitate was isolated by filtration and Soxhlet extracted by THF, ethanol and water. The solid was freeze-dried for 24 h to obtain the final products.

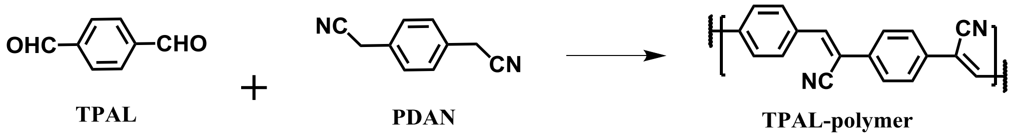

Scheme S4. Synthesis of TPAL-polymer

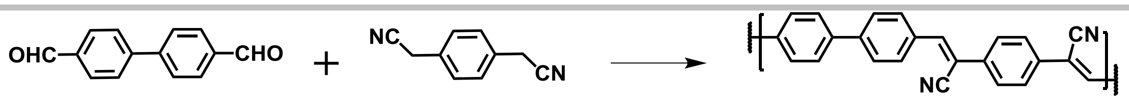

BPDA **PDAN** **BPDA-polymer**

Scheme S5. Synthesis of BPDA-polymer

Structure Simulations

Molecular modeling and Pawley refinement were executed using Materials Studio software package. From the optimized monolayer structures, two stacking modes were investigated, eclipsed (AA), staggered (AB), respectively. All the three COFs of the eclipsed AA stacking modes generated PXRD patterns in good agreement with the experimental data, whereas the staggered AB stacking modes cannot reproduce the experimental data. These optimized structures were further carried out by Pawley refinement using Reflex. The calculated lattice parameters are a = 38.04 Å, b = 37.78 Å, c = 3.68 Å, α = 90°, β = 90° and γ = 120° with R_{WP} = 2.11 %, R_P = 3.21 % for COF-alkene, a = 36.43 Å, b = 36.43 Å, c = 3.71 Å, α = 90°, β = 90° and γ = 120° with R_{WP} = 2.58 %, R_P = 2.06 % for COF-imide and a = 37.29 Å, b= 37.60 Å, c = 3.59 Å, α = 90°, β = 90° and γ = 120° with R_{WP} = 4.87 % and R_P = 2.86 % for COF-imine. The difference plots show that the refined diffraction patterns are consistent with the experimental PXRD data.

Photocatalytic Experiments

Photocatalytic experiments were conducted in a Pyrex top-irradiation reaction vessel connected to a sealed gas circulation system. Hydrogen production was carried out by dispersing 20 mg of powder photocatalyst in an aqueous solution (25 mL) containing triethanolamine (TEOA, 20 vol%) as a sacrificial electron donor and the optimal amount 3 wt % Pt was photodeposited onto the catalyst surface using $H_2PtCl_6{\cdot}6H_2O$ as a platinum precursor. The reaction solution was evacuated three times to remove air thoroughly before irradiation under a 300 W Xenon lamp (Microsolar 300, PerfectLight, Beijing) with a 420 nm cut-off filter. The reaction vessel was kept at room temperature by the cooling of circulating water during the reaction. The evolved gases were analyzed by gas chromatography (GC-2014ATF, Shimadzu) with a known concentration of hydrogen as standard gas.

The apparent quantum efficiency (AQE) for hydrogen production was measured under illumination of a 300 W Xe lamp with different bandpass filters. The reported AQE values here are maximum attainable results after varying the amounts of photocatalysts used. Depending on the amount of hydrogen evolution during an average of one hour photocatalytic reaction, the AQE was calculated based on the equation below:

$$\mathrm{AQE} = \frac{N_e}{N_p} \times 100\% = \frac{2 \times M \times N_A \times h \times c}{S \times P \times t \times \lambda} \times 100\%$$

Where N_p is the total incident photons, N_e is the total reactive electrons, M is the amount of hydrogen molecules, N_A is Avogadro constant, h is the Planck constant, c is the speed of light, S is the irradiation area, P is the intensity of irradiation light, t is the photoreaction time, λ is the wavelength of the monochromatic light.

Mott-Schottky Plot Measurements

Mott-Schottky plots were measured on a CHI 760E instrument (Shanghai, China) in a standard three-electrode system in 0.1 M Na_2SO_4 aqueous solution with the photocatalyst-coated glassy carbon as the work electrode, a platinum foil as the counter electrode, and an Ag/AgCl (saturated KCl solution) as the reference electrode at frequencies of 2k, 5k and 10k Hz, respectively. The catalyst (4 mg) was dispersed in 1.5 mL ethanol with addition of 10 μL 5 wt% Nafion solution, after sonication for 5 minutes, the work electrodes were prepared by dropping the suspension (20 μL) onto the surface of glass carbon electrode (0.196 cm^{-2}).

Femtosecond Transient Absorption (fs-TA) Experiments

The fs-TA spectroscopy was obtained on Helios (Ultrafast Systems LLC) spectrometers based on a regeneratively amplified Ti-Sapphire laser source (Coherent Legent, 800 nm, 150 fs, 5 mJ $pulse^{-1}$, 1 kHz repetition rate) in a transmission mode. Portion of the 800 nm output (75 %) pulse was frequency-doubled in a BaB_2O_4 crystal, which could generate 400 nm pump light, meanwhile, the remaining portion of the output was concentrated into a sapphire window to produce continuous white light (500 – 780 nm) as probe light. The energy of 400 nm pump beam was formed from part of the 800 nm output pulse from amplifier and the power was adjusted to be 100μJ cm^{-2} by neutral density optical filters. The probe spectra (reference) could be split from the continuous white light and sent into Helios spectrometers. Then transient absorption changes were tested in the visible region from 500 to 780 nm. The solution was placed in a 1 mm optical path length quartz cuvette. Experimental measurements were performed at room temperature. For each measurement, the pump-probe delay scan was repeated two times to give the averaged experiment data. The transient spectroscopy samples were prepared via dispersing three COF catalysts in water with a concentration of 0.1 mg mL^{-1} and unltrasonicated to enhance dispersibility.

Calculation of Transmission Function

The transmission function is calculated by Dmol3 package in Materials Studio of Accelrys Inc, in which Keldysh nonequilibrium Green's function (NEGF) method[6, 7] is applied with density-functional theory (DFT) to evaluate the electron transport properties of nanoscale devices. The generalized gradient approximation (GGA) with the Perdew-Burke-Ernzerhof (PBE) exchange-correlation functional was used in this simulation and all-electron double numerical basic set was applied with polarization functions (DNP). The convergence tolerances of energy, force, and displacement were set to 1×10^{-5} Ha, 2.0×10^{-3} Ha/Å, and 5.0×10^{-3} Å, respectively.

Water Contact Angle Measurements

Static water contact angles were tested by applying a drop shape analysis system-Kruss DSA 100. The powder samples were tested by pressed into thin discs in a stainless steel spacer with controlled mass amounts. 3 μL water were automatically dropped on the surface of the samples. The contact angle between a drop and the sample was recorded from an image of the drop taken by a digital camera and calculated by the software of Drop Shape Analysis.

Results and Discussion

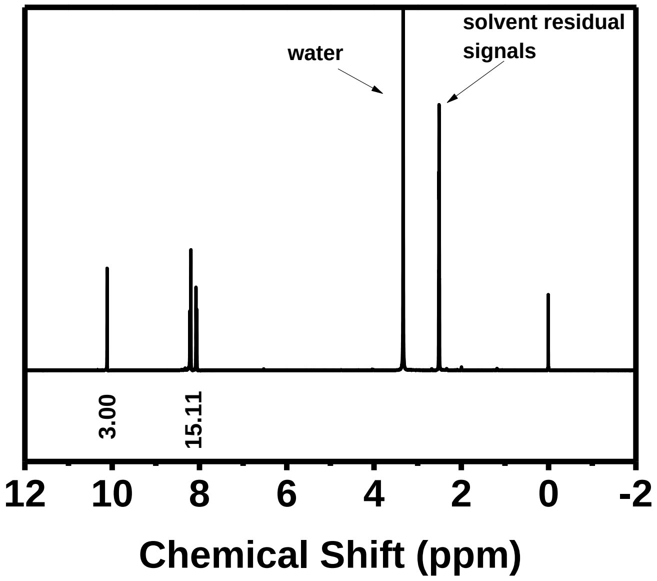

Figure S1. 1H NMR ($(CD_3)_2SO$, 40MHz) spectrum of TFPB. All the chemical shifts at 10.10 (s. 3H, -CHO), 8.24-8.00 (m, 15H, -ArH) ppm demonstrate the structure of TFPB.

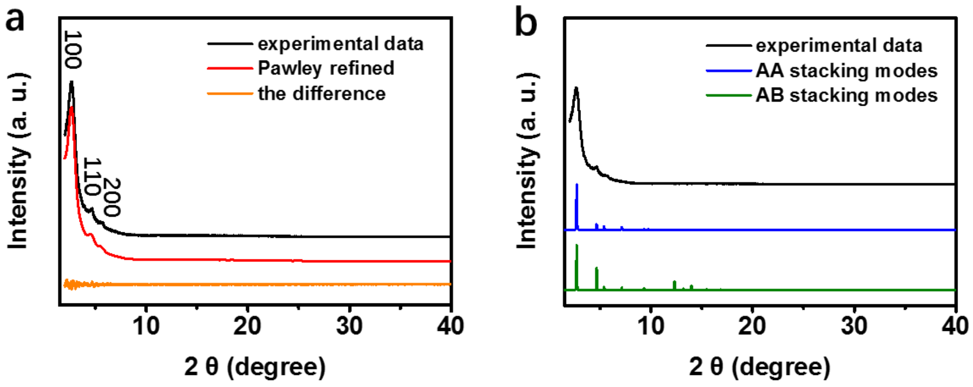

Figure S2. PXRD profiles of COF-alkene: the experimental data (black), Pawley refined (red) and their difference (orange), simulated using the AA stacking modes (blue) and AB stacking modes (green). The Pawley refinement yields a PXRD pattern that is in good agreement with the experimentally observed pattern with low R_{WP} = 2.11 % and R_P = 3.21 %.

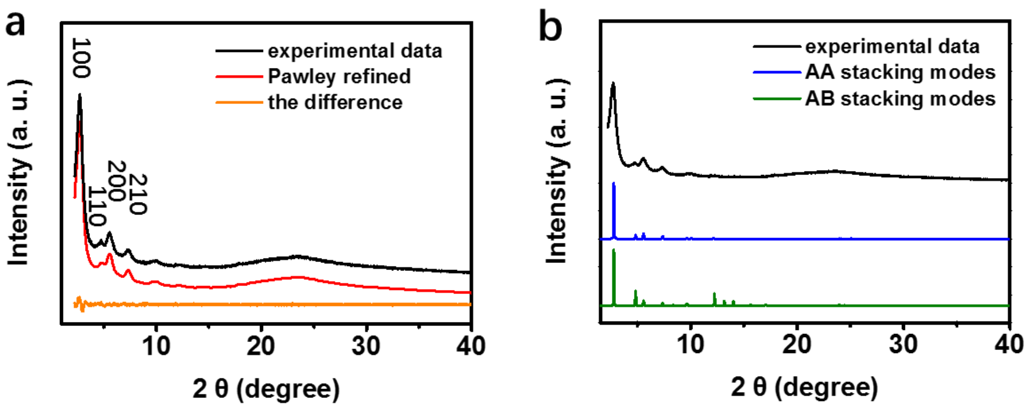

Figure S3. PXRD profiles of COF-imide: the experimental data (black), Pawley refined (red) and their difference (orange), simulated using the AA stacking modes (blue) and AB stacking modes (green). The Pawley refinement yields a PXRD pattern that is in good agreement with the experimentally observed pattern with low R_{WP} = 2.56 % and R_P = 2.06 %.

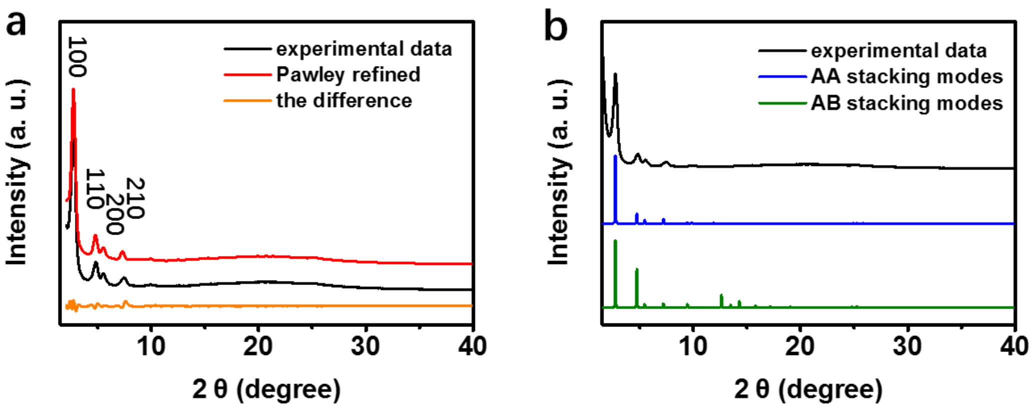

Figure S4. PXRD profiles of COF-imine: the experimental data (black), Pawley refined (red) and their difference (orange), simulated using the AA stacking modes (blue) and AB stacking modes (green). The Pawley refinement yields a PXRD pattern that is in good agreement with the experimentally observed pattern with R_{WP} = 4.87 % and R_P = 2.86 %.

The PXRD patterns reveal that all three products are highly crystalline polymers. For COF-alkene, the PXRD peaks at 2.68°, 4.65° and 5.36° are assigned to the (100), (110) and (200) facets, respectively. In the case of COF-imide, the PXRD peaks appear at 2.77°, 4.82°, 5.54° and 7.35°, which are attributed to the (100), (110), (200) and (210) facets, respectively. The COF-imine exhibits the PXRD peaks at 2.73°, 4.73°, 5.47° and 7.22°, which are assigned to the (100), (110), (200) and (210) facets, respectively.

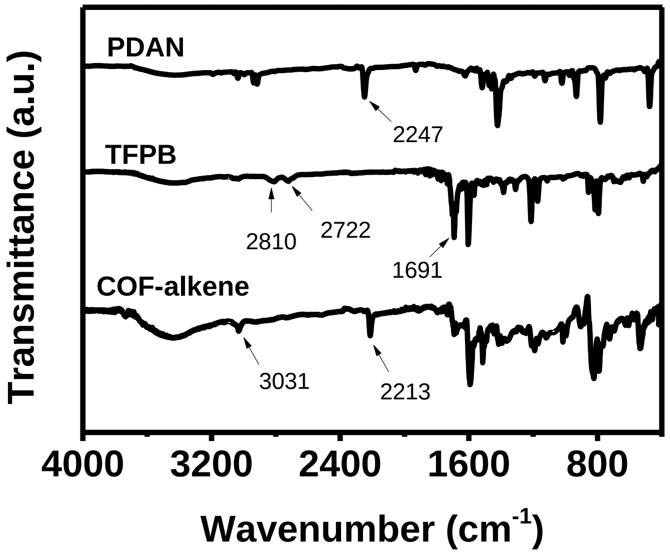

Figure S5. FT-IR spectra of COF-alkene and the monomers. With respect to two monomers, the newly appeared peaks at 2213 cm^{-1} and 3031 cm^{-1} for COF-alkene correspond to the cyano side group (C=C-CN) and the C-H stretching vibration of C=CH, respectively. Furthermore, the signal at 2722 cm^{-1} assigned to the C-H bond of the aldehyde group of monomer disappears. All these strongly indicate the polycondensation between TFPB and PDAN.

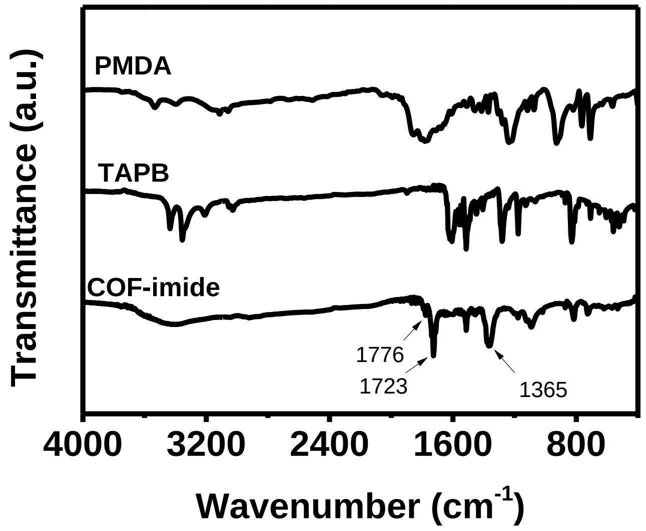

Figure S6. FT-IR spectra of COF-imide and the monomers. The FT-IR spectra for COF-imide show two characteristic absorption peaks at 1776 and 1723 cm^{-1}, corresponding to asymmetric and symmetric vibrations of C=O groups of the five-membered imide rings, whereas the peak at 1376 cm^{-1} is attributed to the stretching vibration of C-N-C moiety.

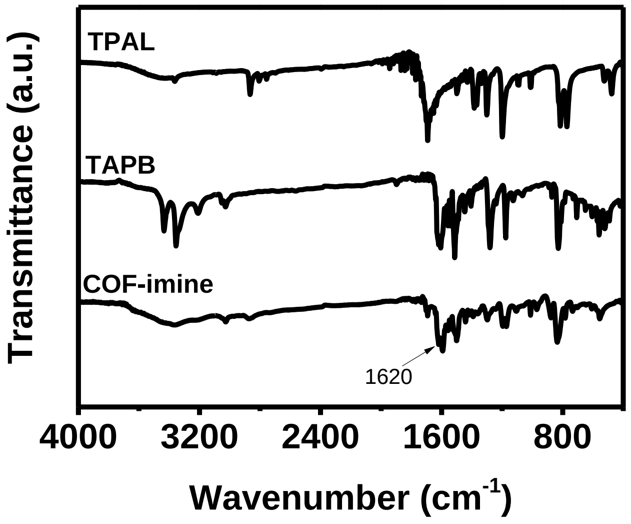

Figure S7. FT-IR spectra of COF-imine and the monomers. A stretching vibration band at 1620 cm^{-1} for COF-imine is assigned to the C=N bond.

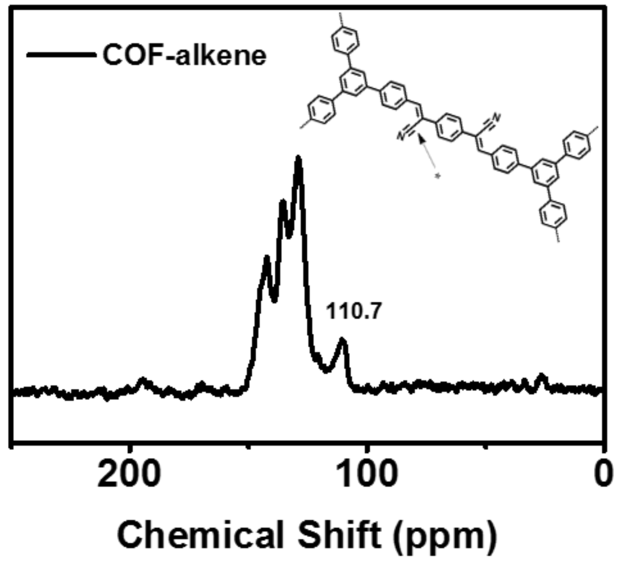

Figure S8. Solid-state ^{13}C CP/MAS NMR spectrum of COF-alkene. The peak at 110.7 ppm confirms the existence of cyano side groups.

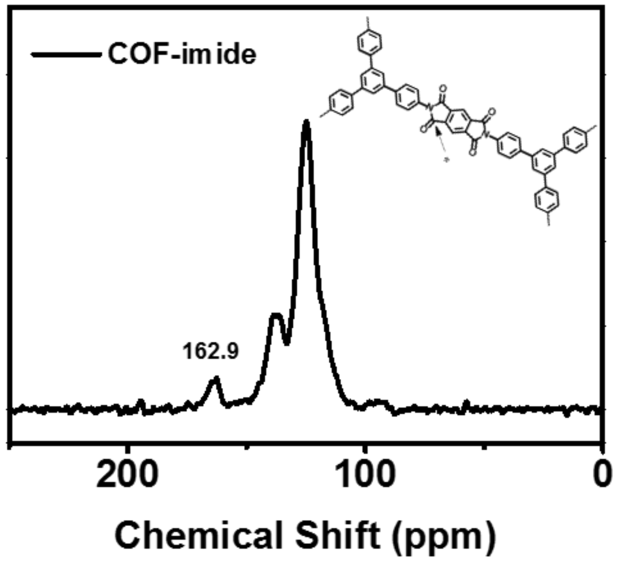

Figure S9. Solid-state ^{13}C CP/MAS NMR spectrum of COF-imide. The peak at 162.9 ppm confirms the presence of carbonyl carbon of the imide ring.

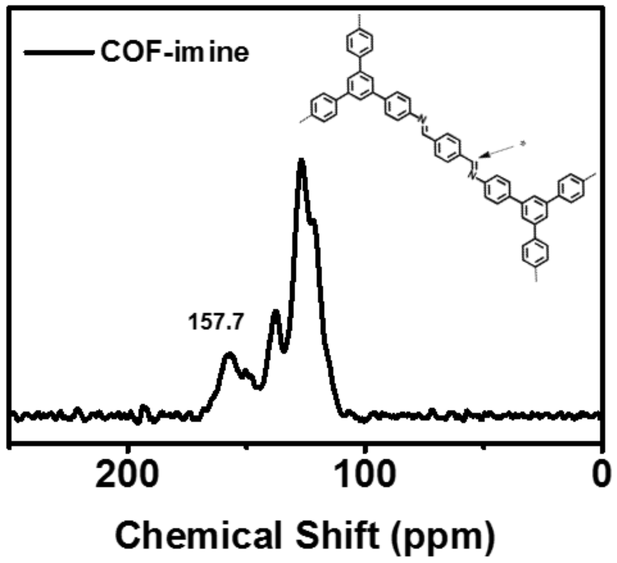

Figure S10. Solid-state ^{13}C CP/MAS NMR spectrum of COF-imine. The peak at 157.7 ppm is assigned to the carbon of C=N bond.

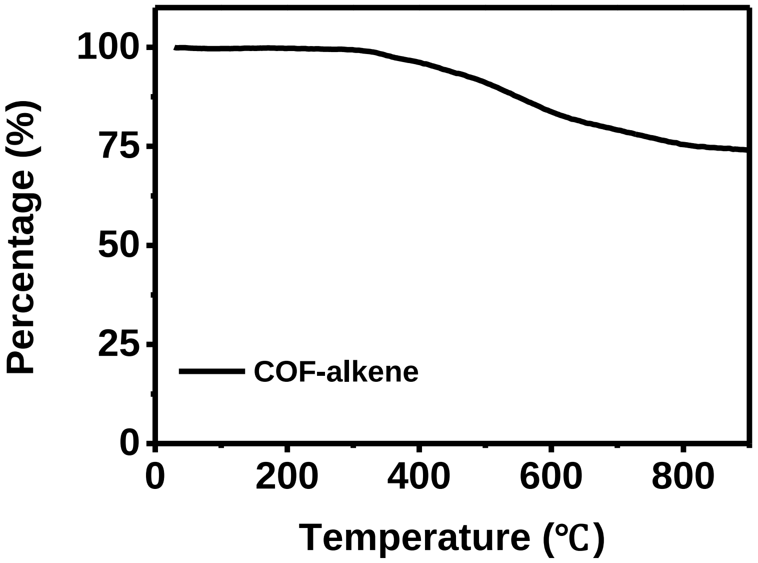

Figure S11. TGA curve of COF-alkene. The curve exhibits no significant weight loss of COF-alkene until 420 ℃.

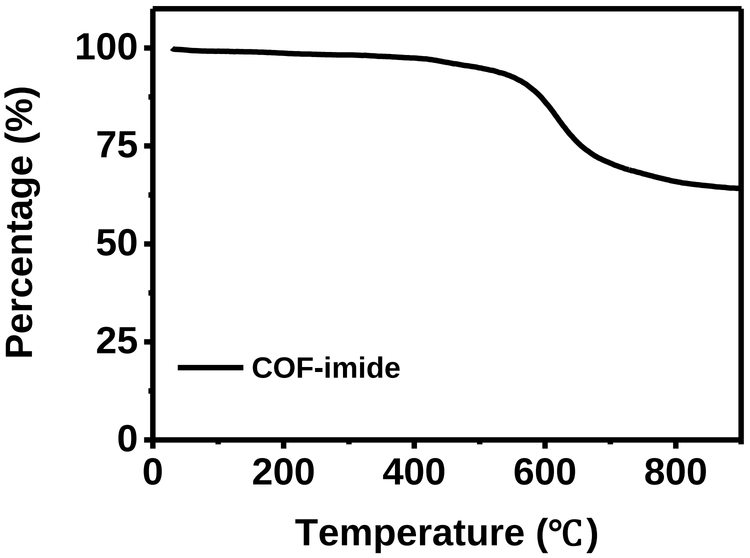

Figure S12. TGA curve of COF-imide. The curve exhibits no significant weight loss of COF-imide until 520 ℃.

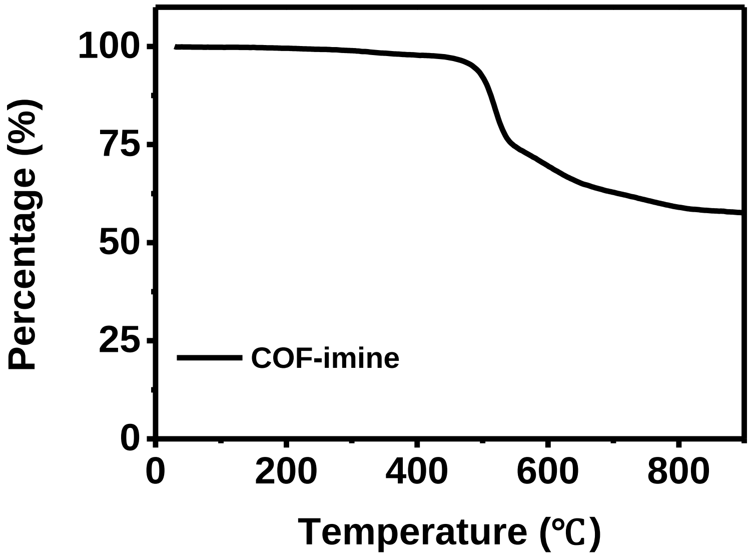

Figure S13. TGA curve of COF-imine. The curve exhibits no significant weight loss of COF-imine until 480 ℃.

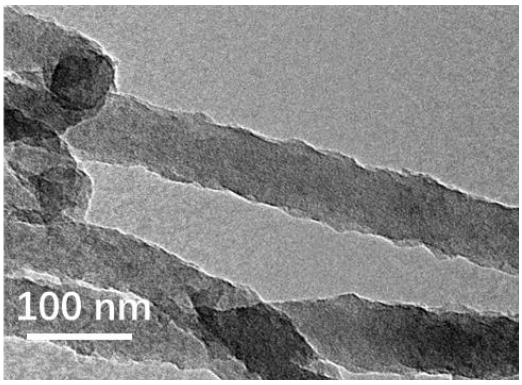

Figure S14. TEM image of COF-alkene.

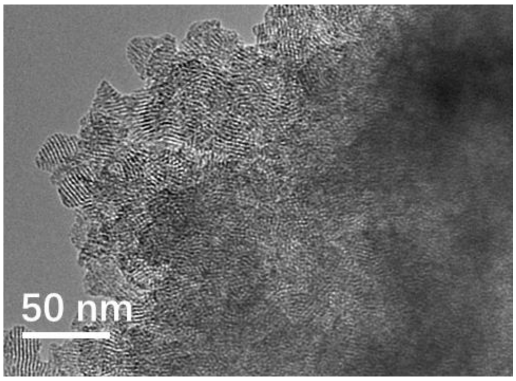

Figure S15. TEM image of COF-imide.

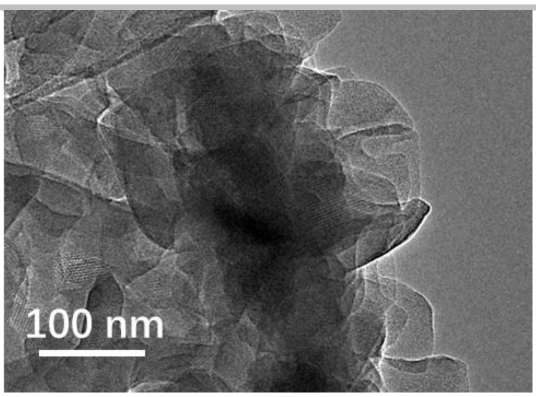

Figure S16. TEM image of COF-imine.

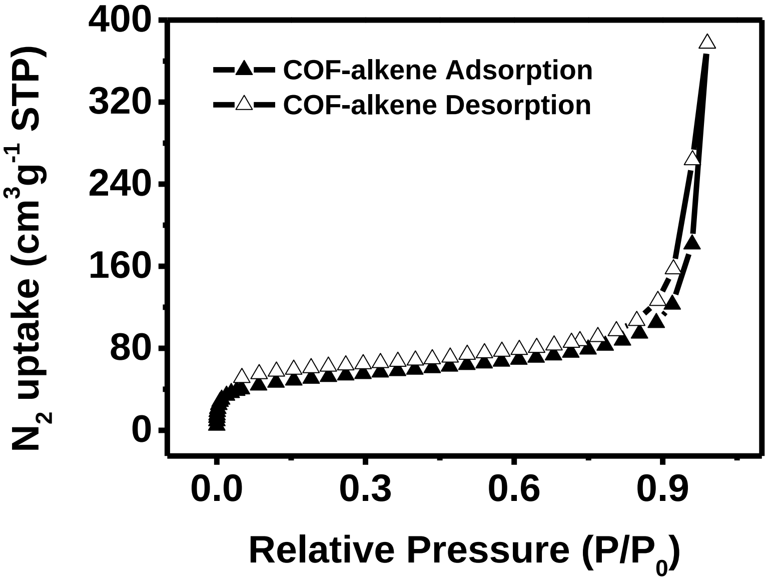

Figure S17. Nitrogen sorption isotherm curve of COF-alkene. The COF-alkene was synthesized similar to the literature-reported 2DPPV [2]. The BET surface area for COF-alkene is calculated to be 185 m^2g^{-1}, which is lower than that computationally predicted for the atomistic model of a perfectly crystalline structure (2160 m^2g^{-1}), but is commonly discovered for some other sp^2 carbon-linked COF such as 472 m^2g^{-1} for 2DPPV, 317 m^2g^{-1} for 2D CCP-HATN [8a], 322 m^2g^{-1} for sp^2c-COF-2[8b], 232 m^2g^{-1} for TP-COF[8c].

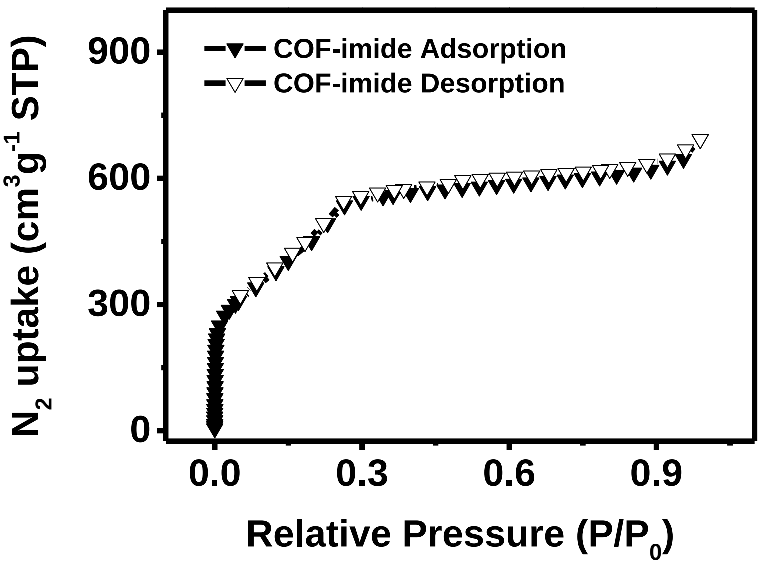

Figure S18. Nitrogen sorption isotherm curve of COF-imide. The COF-imide was synthesized similar to the literature-reported PI-COF-2 [4]. The BET surface area for COF-imide is calculated to be 1664 m^2g^{-1}, which is larger than that of PI-COF-2 with 1297 m^2g^{-1}.

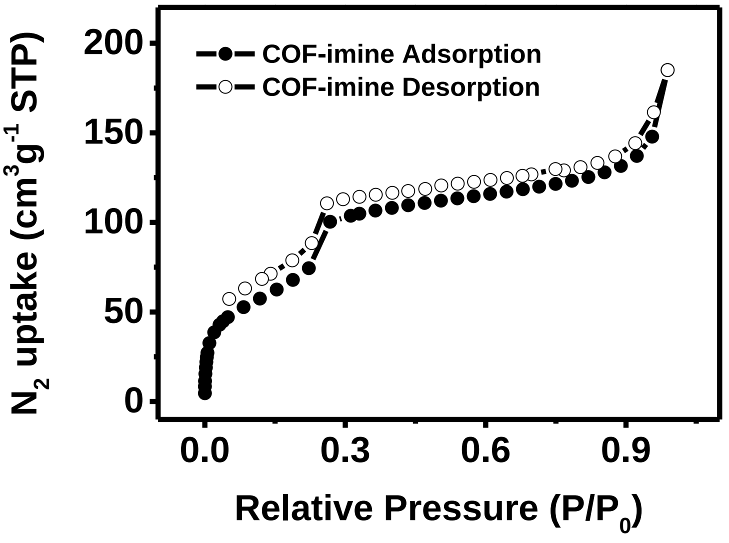

Figure S19. Nitrogen sorption isotherm curve of COF-imine. The COF-imine was synthesized similar to the literature-reported TPB-TP-COF [5]. The BET surface area for COF-imine is calculated to be 248 $m^2\,g^{-1}$, which is larger than that of TPB-TP-COF with 16 $m^2\,g^{-1}$.

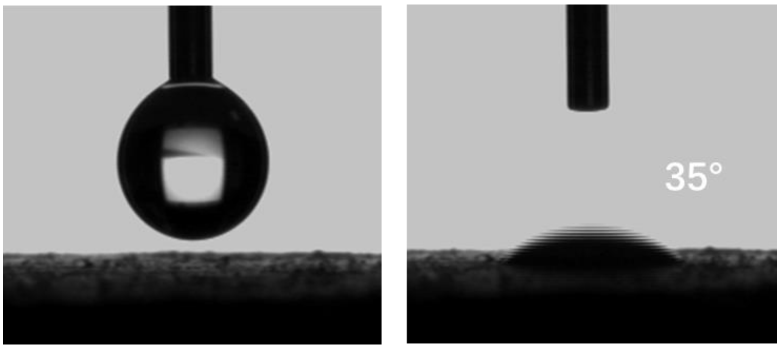

Figure S20. Water contact angle measurements of COF-alkene at room temperature in air.

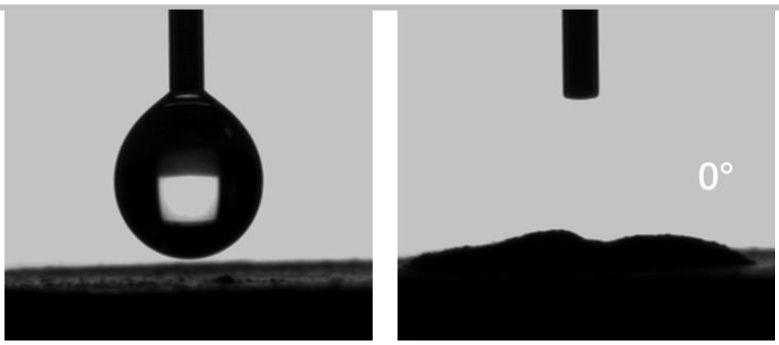

Figure S21. Water contact angle measurement of COF-imide at room temperature in air. The pressed pellet of COF-imide began to swell once absorbing the water drop, and the component and crystallinity of COF-imide are still perserved.

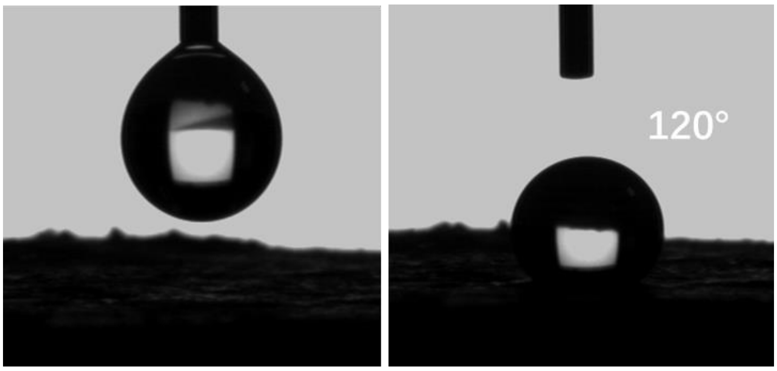

Figure S22. Water contact angle measurements of COF-imine at room temperature in air.

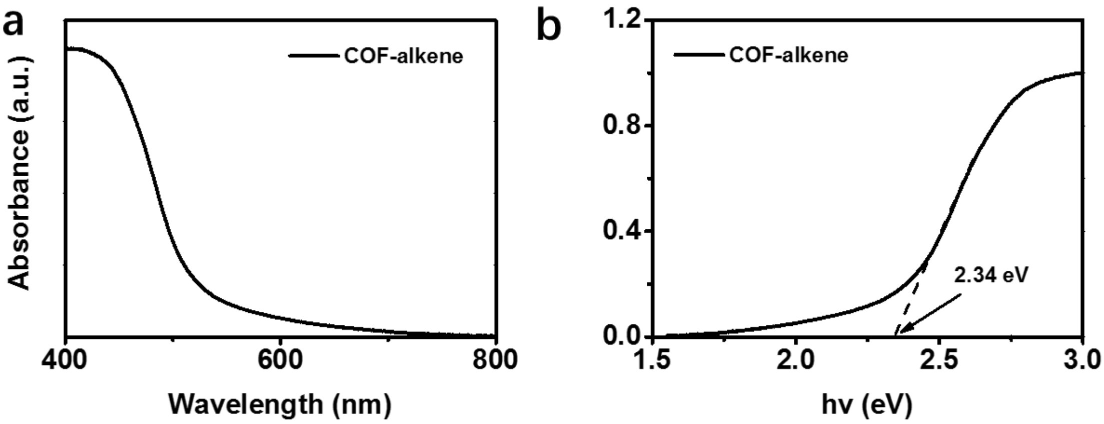

Figure S23. a) UV-Vis DRS spectrum of the COF-alkene and b) the corresponding Tauc plots, the intersection of the dashed lines with x-axis indicates the value of optical bandgap.

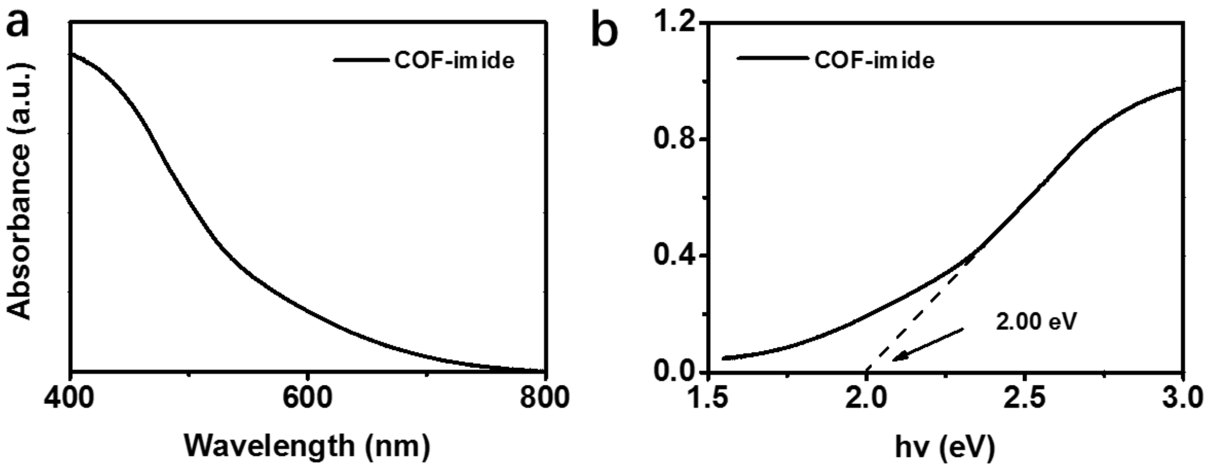

Figure S24. a) UV-Vis DRS spectrum of the COF-imide and b) the corresponding Tauc plots, the intersection of the dashed lines with x-axis indicates the value of optical bandgap.

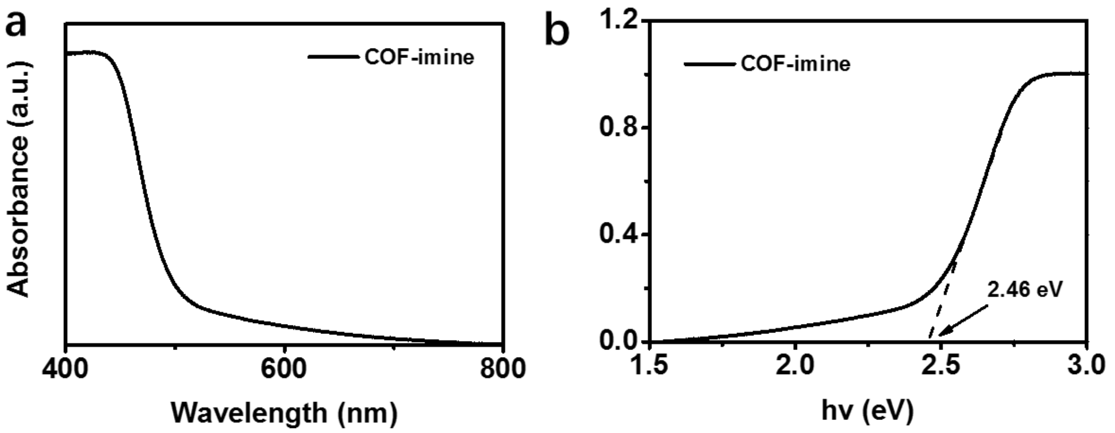

Figure S25. a) UV-Vis DRS spectrum of the COF-imine and b) the corresponding Tauc plots, the intersection of the dashed lines with x-axis indicates the value of optical bandgap.

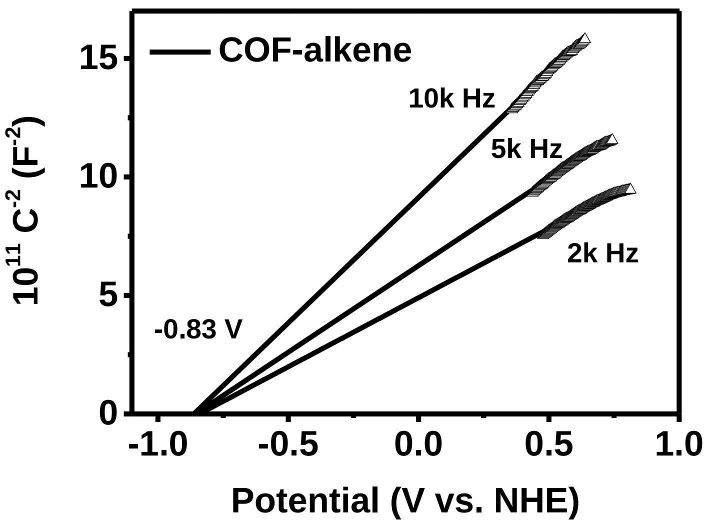

Figure S26. Mott-Schottky plots for COF-alkene in 0.1M Na_2SO_4 aqueous solution.

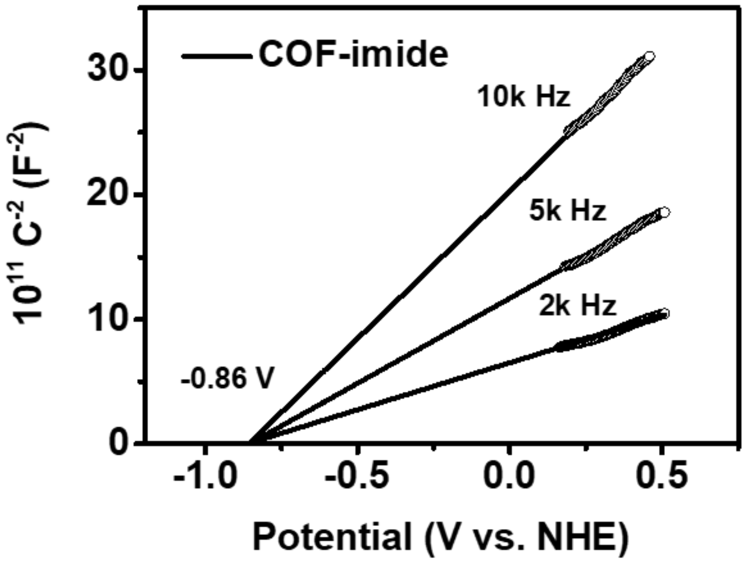

Figure S27. Mott-Schottky plots for COF-imide in 0.1M Na_2SO_4 aqueous solution.

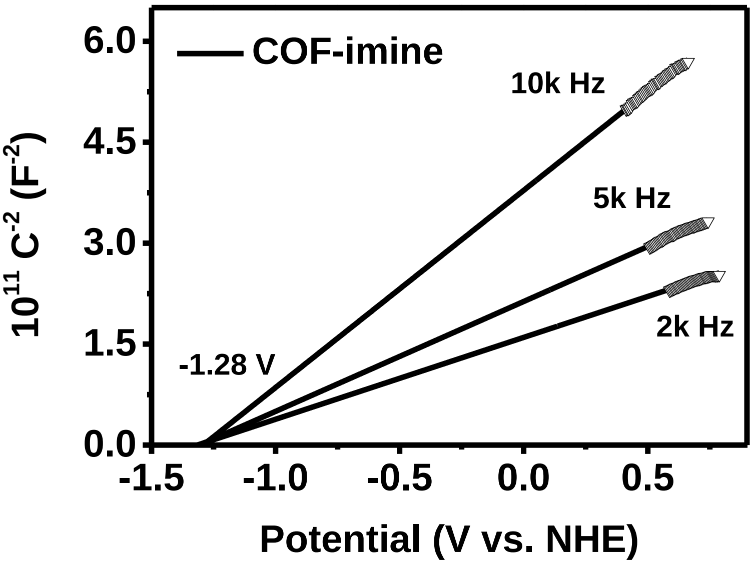

Figure S28. Mott-Schottky plots for COF-imine in 0.1M Na_2SO_4 aqueous solution.

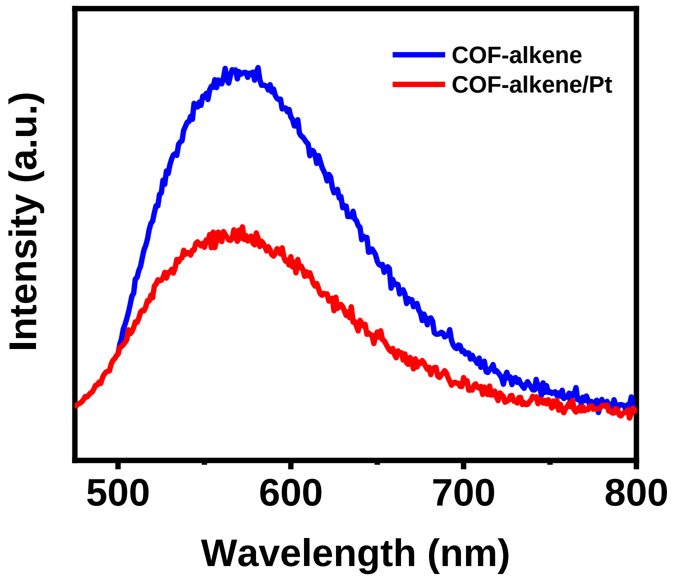

Figure S29. Steady-state photoluminescence spectra of COF-alkene and COF-alkene/Pt. COF-alkene/Pt exhibits much lower emission intensity with respect to COF-alkene, indicating much weaker charge recombination rate when Pt deposited on the surface of COF-alkene.

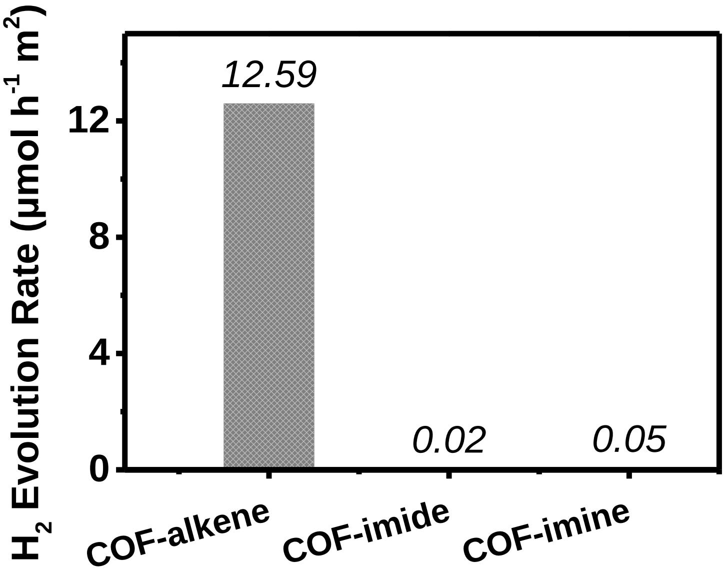

Figure S30. PHE activities of three COF catalysts normalized to the corresponding BET surface area.

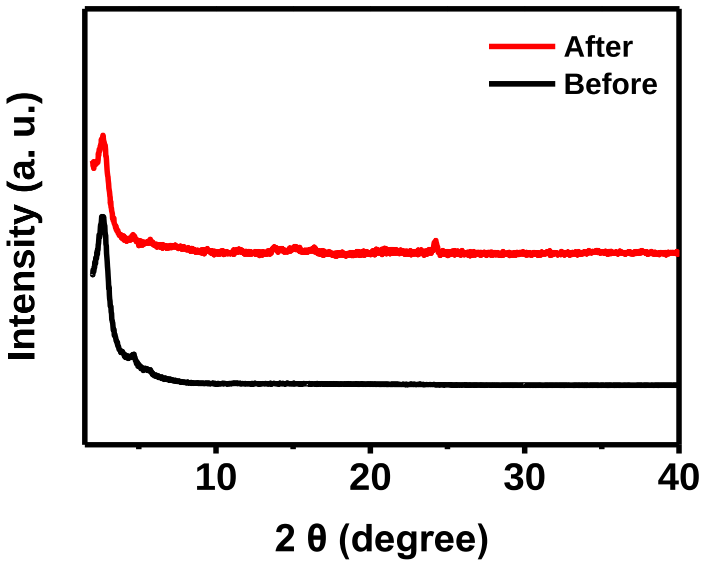

Figure S31. PXRD characterization of COF-alkene before and after photocatalytic experiment. We can see that COF-alkene still maintains long-range ordering after photocatalytic experiments.

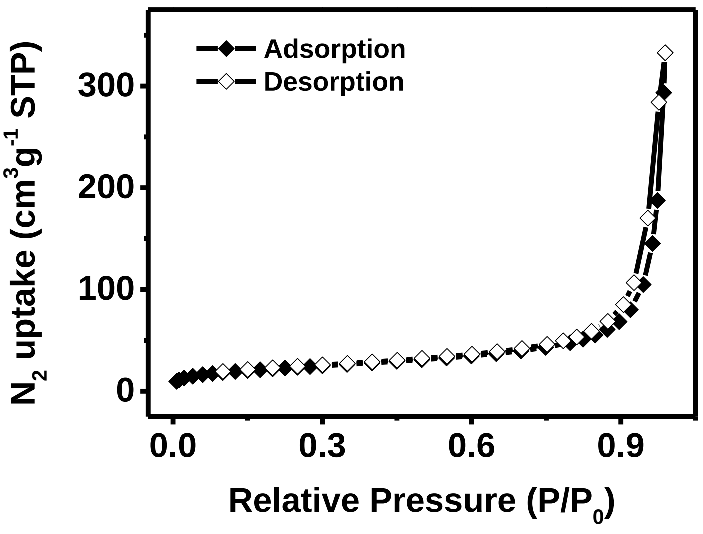

Figure S32. Nitrogen sorption test of COF-alkene after photocatalytic experiment. The BET surface area for COF-alkene after PHE experiment is calculated to be 81 m^2g^{-1}, a little bit lower than that of origin COF-alkene. This is because the Pt nanoparticles deposited on the surface of COF will increase the mass of sample and block the pore channels, thus resulting in reduction of BET surface area, the same with the literature previously reported [11, 15].

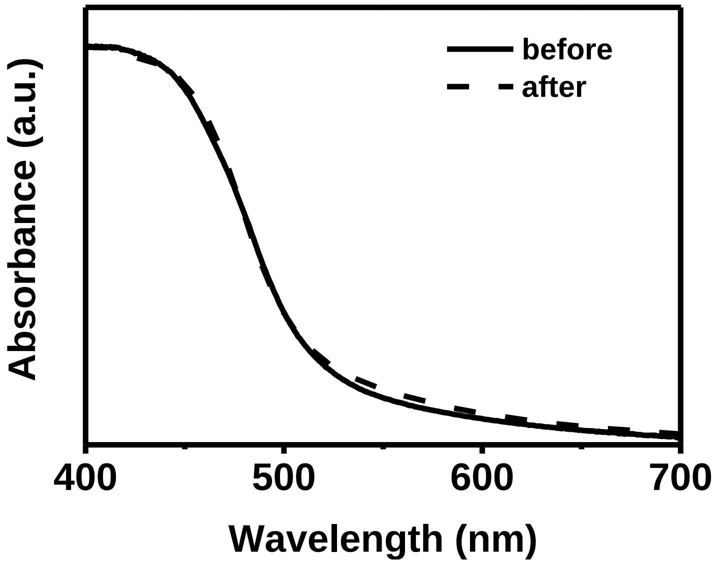

Figure S33. UV-vis DRS characterization of COF-alkene before and after photocatalytic experiment. We can see that UV-vis DRS remains almost unchanged after photocatalytic experiment.

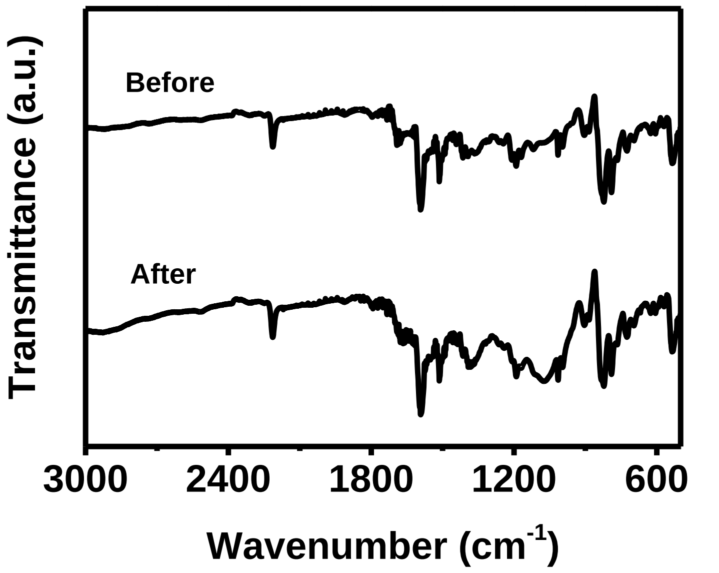

Figure S34. FT-IR characterization of COF-alkene before and after photocatalytic experiment. We can see that COF-alkene well preserves the COF structure integrity after photocatalytic experiment.

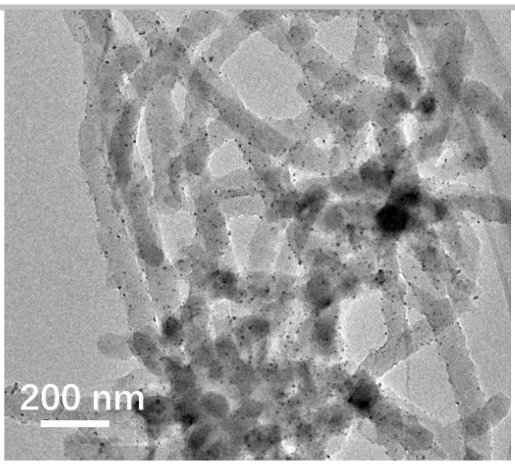

Figure S35. TEM image of COF-alkene after photocatalytic experiment. It shows that Pt nanoparticles was in-situ photoreduced and deposited on the surface of COF and the fiber-like morphology of COF-alkene remain nearly unchanged.

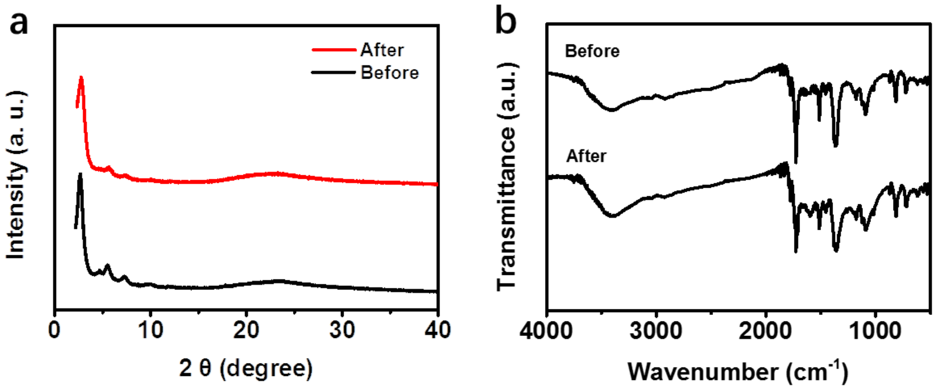

Figure S36. a) PXRD and b) FT-IR characterization of COF-imide before and after photocatalytic experiment of 5 h. We can see that COF-imine well preserves the COF long-range ordering and structure integrity after photocatalytic experiment

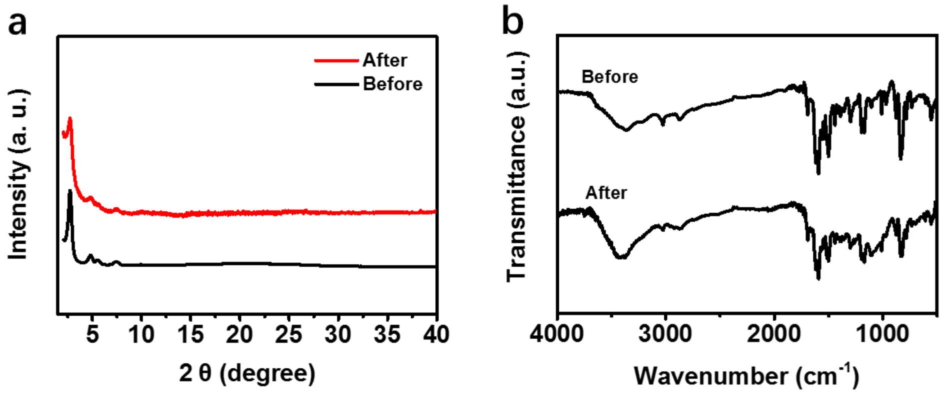

Figure S37. a) PXRD and b) FT-IR characterization of COF-imine before and after photocatalytic experiment of 5 h. We can see that COF-imine well preserves the COF long-range ordering and structure integrity after photocatalytic experiment

As exhibited in Figure S36-37, the COF-imide and COF-imine still maintained the composition and long-range ordering after PHE experiment, so we reason that COF-imide and COF-imine did not decompose in this process within 5 h.

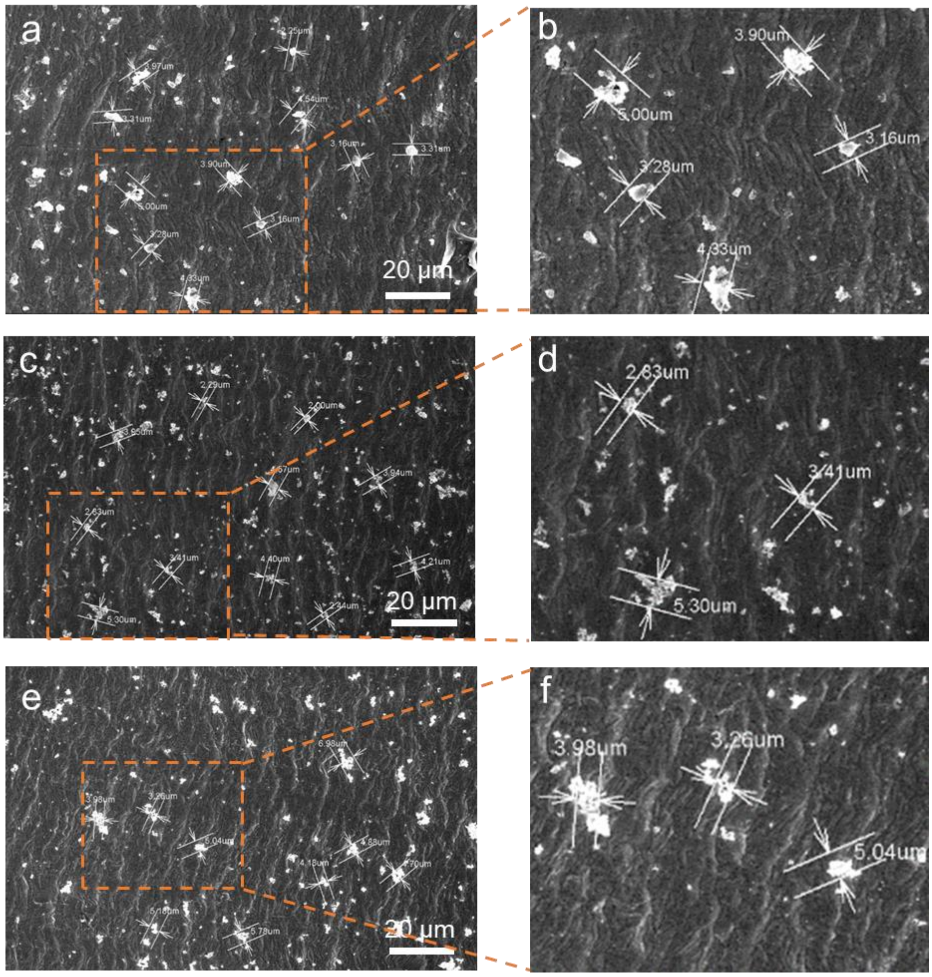

Figure S38. Ex-situ SEM images of (a, b) COF-alkene, (c, d) COF-imide, (e, f) COF-imine under photocatalytic conditions. As we can see that all the three COFs were detected as aggregated particles with sized ranging from 2 to 7 µm**.**

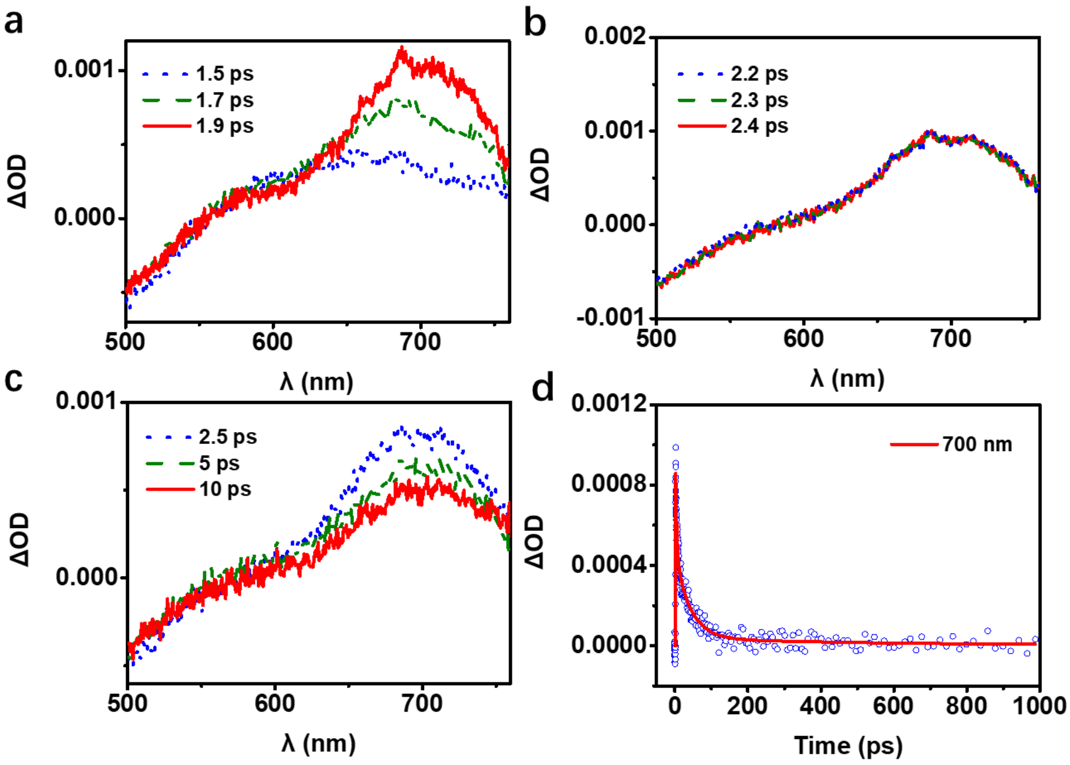

Figure S39. The fs-TA spectra of COF-alkene in 0.1 mg mL^{-1} aqueous solution without TEOA obtained following a 400 nm laser pulse from (a) 1.5 ps to 1.9 ps, (b) 2.2 ps to 2.4 ps, and (c) 2.5 ps to 10 ps. (d) The kinetics of the characteristic fs-TA absorption bands observed at 700 nm.

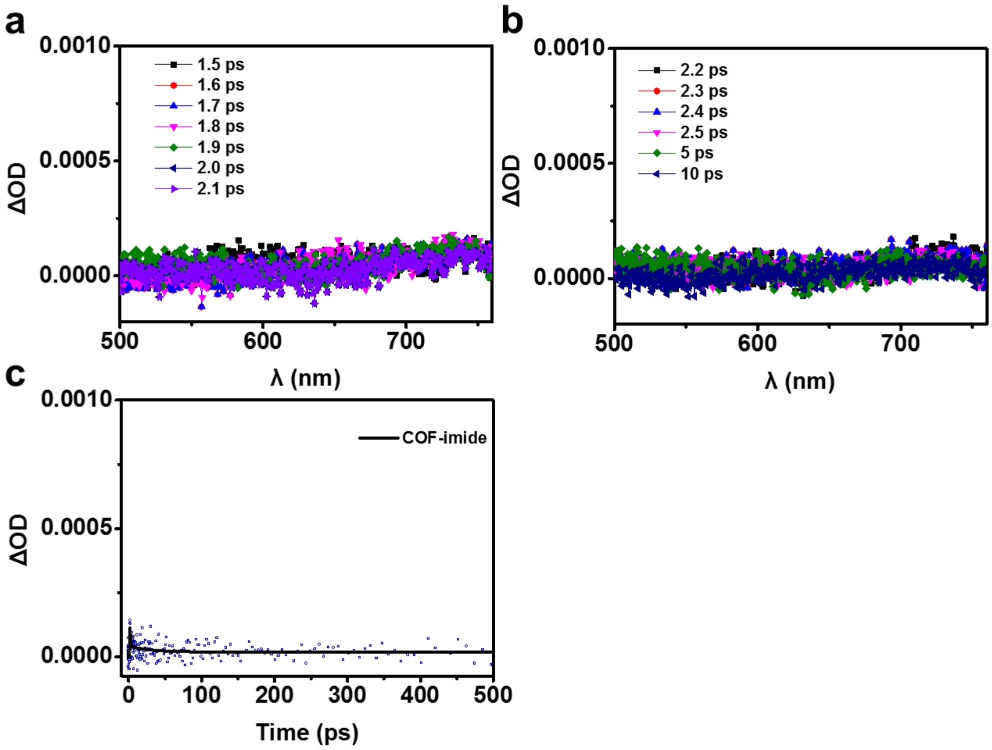

Figure S40. The fs-TA spectra of COF-imide in 0.1 mg mL^{-1} aqueous solution without TEOA with obtained following a 400 nm laser pulse from (a) 1.5 ps to 2.1 ps and (b) 2.2 ps to 10 ps. (f) The kinetics of the characteristic fs-TA absorption bands of COF-imide.

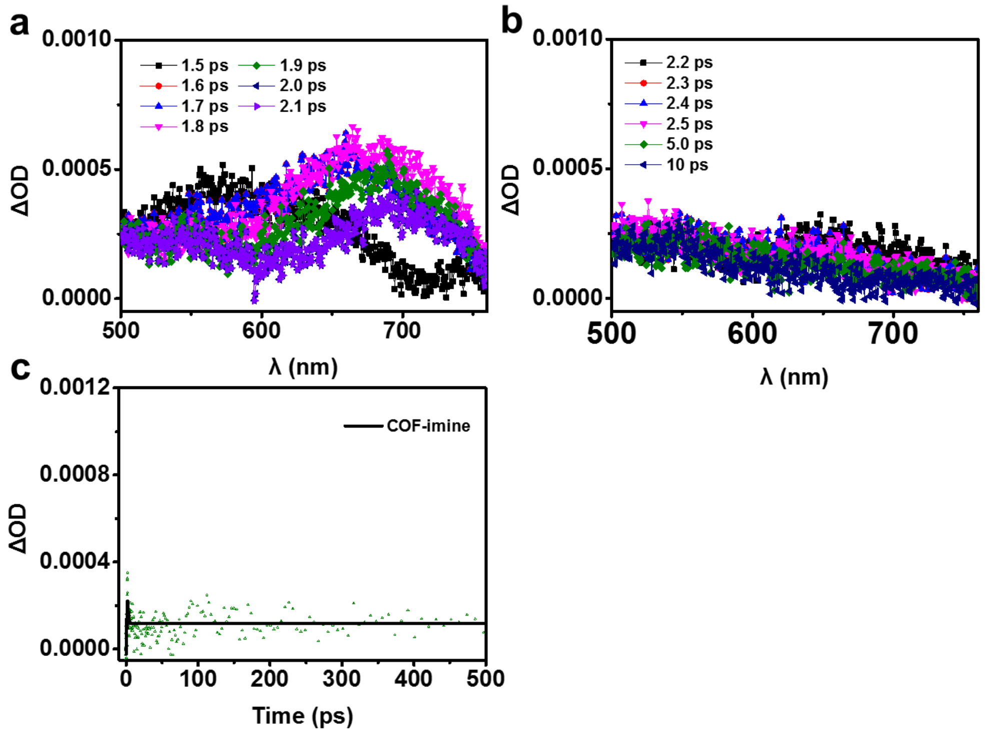

Figure S41. The fs-TA spectra of COF-imine in 0.1 mg mL^{-1}aqueous solution without TEOA obtained following a 400 nm laser pulse from (a) 1.5 ps to 2.1 ps and (b) 2.2 ps to 10 ps. (f) The kinetics of the characteristic fs-TA absorption bands of COF-imine.

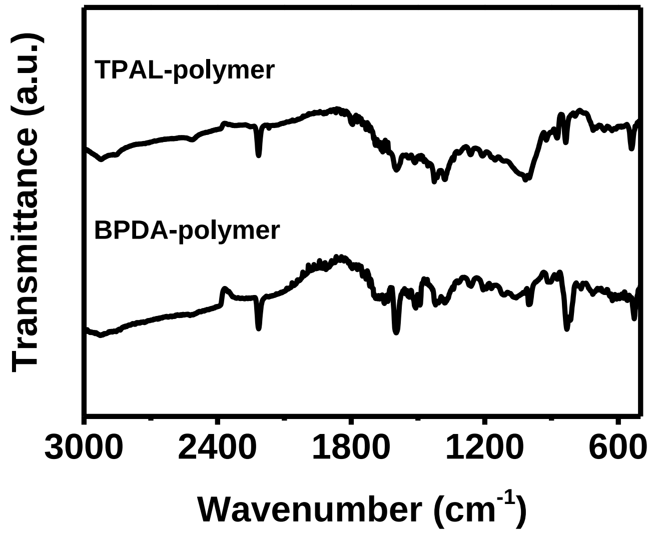

Figure S42. FT-IR spectra of TPAL-polymer and BPDA-polymer. The peaks at 2214 cm^{-1} for TPAL-polymer and 2216 cm^{-1} for BPDA-polymer correspond to the cyano side group (C=C-CN).

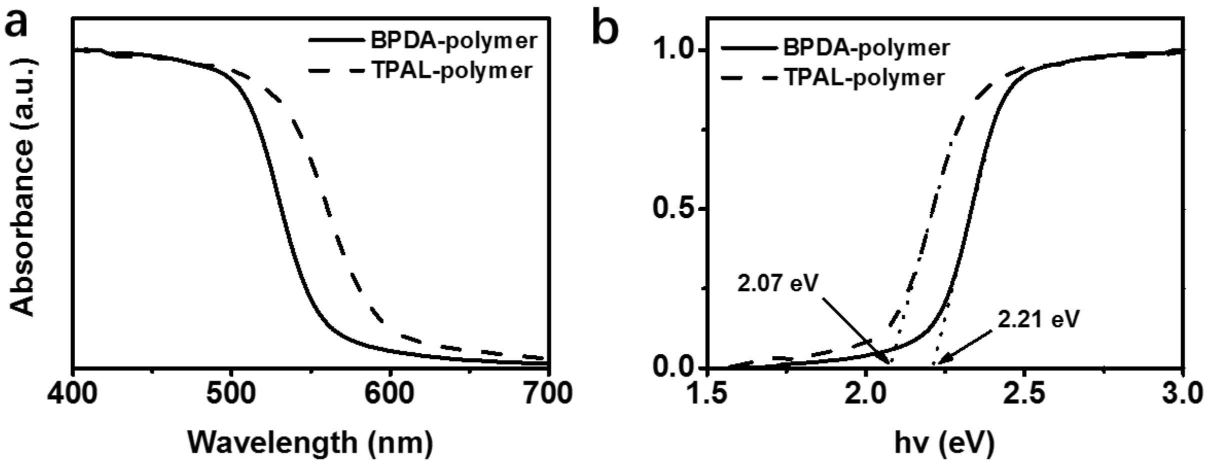

Figure S43. a) UV-Vis DRS spectra of the BPDA-polymer and TPAL-polymer and b) the corresponding Tauc plots, the intersection of the dashed lines with x-axis indicates the value of optical bandgap.

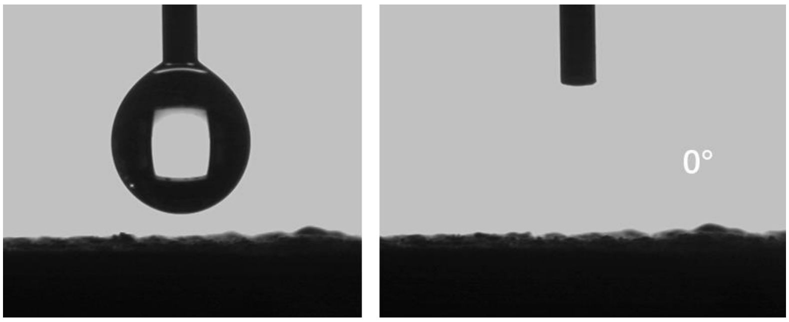

Figure S44. Water contact angle measurements of TPAL-polymer at room temperature in air.

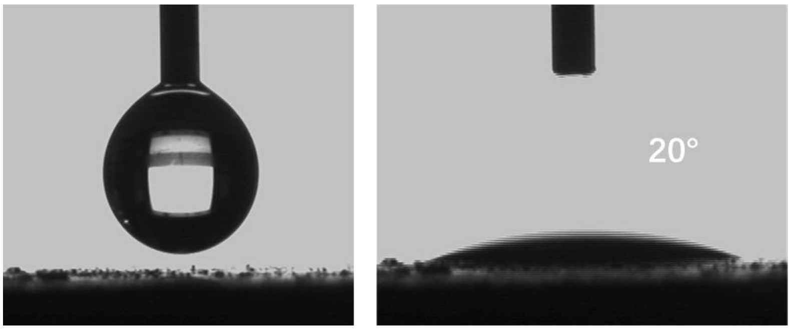

Figure S45. Water contact angle measurements of BPDA-polymer at room temperature in air.

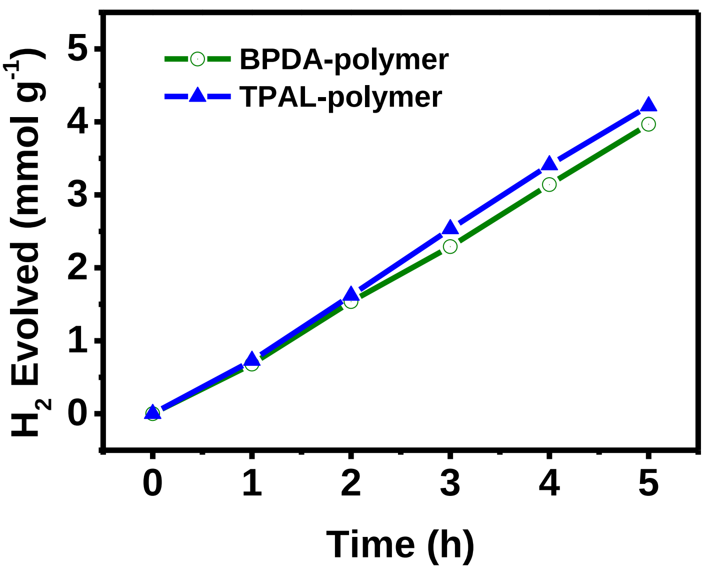

Figure S46. PHE activities of BPDA-polymer and TPAL-polymer catalysts under visible light-irradiation (λ > 420 nm) using TEOA as sacrificial agent and Pt as co-catalyst.

Table S1. The difference of COF-alkene of this work and 2DPPV of the literature reported.[2]

COFs	COF-alkene	2DPPV
Monomers	0.25 mmol TFPB, 0.4 mmol PDAN	0.384 mmol PDAN, 0.256 mmol TFPB
Solvent	mesitylene/1,4-dioxane (8 mL, 1/5 by vol.)	o-dichlorobenzene (4 mL)
Catalysts	NaOH solution (1.6 mL, 6 M)	Cs_2CO_3 (250 mg, 115 mmol)
Temperature	90 ℃	100 ℃
Morphology	Fiber-like	Sheet-like
Optical band gap	2.34 eV	2.1 eV

Table S2. The difference of COF-imide of this work and PI-COF-2 of the literature reported.[4]

COFs	COF-imide	PI-COF-2
Monomers	0.1 mmol TAPB, 0.15 mmol PMDA	0.1 mmol TAPB, 0.15 mmol PMDA
Solvent	2 mL NMP/2 mL mesitylene/0.2 mL water	0.5 mL mesitylene/0.5 mL NMP
Catalysts	0.4 mL isoquinoline	0.05 mL isoquinoline
Temperature	200 ℃	200 ℃

Table S3. The difference of COF-imine of this work and TPB-TP-COF of the literature reported.[5]

COFs	COF-imine	TPB-TP-COF
Monomers	0.2 mmol TAPB, 0.24 mmol TPAL	0.08 mmol TAPB, 0.12 mmol TPAL
Solvent	4 mL o-DCB/4 mL n-butanol	0.5 mL o-DCB/0.5 mL n-butanol
Catalysts	1 mL 6 M aqueous acetic acid	0.1 mL 6 M aqueous acetic acid
Temperature	120 ℃	120 ℃

Table S4. Representative summary of photocatalytic H_2 evolution AQE performance of literature reported covalent organic frameworks or polymers.

Catalysts		Conditions	Sacrificial Electron Donor	Amount of Catalysts (mg)	AQE (%)	Refers
COFs	**COF-alkene**	**>420 nm**	**TEOA**	**20**	**6.7 (420 nm)**	**This Work**
	N_2-COF (azine-linked)	AM 1.5	TEOA	5	0.16	[9] *J. Am. Chem, Soc*. **2017**, *139*, 16228-16234.
	TP-BDDA (β-ketoenamine-linked)	>395 nm	TEOA	10	1.3 (420 nm)	[10] *J. Am. Chem. Soc*. **2018**, *140*, 1423-1427.
	TFPT-COF (hydrazone-linked)	>420 nm	TEOA	4	2.2 (400 nm)	[11] *Chem. Sci*. **2014**, *5*, 2789-2793.
	N_3-COF (azine-linked)	>420 nm	TEOA	5	0.44 (450 nm)	[12] *Nat. Commun*. **2015**, *6*, 8508.
	FS-COF (β-ketoenamine-linked)	>420 nm	ascorbic acid	5	3.2 (420 nm)	[13] *Nat. Chem.* **2018**, *10*, 1180-1189.
	sp^2c-COF	>420 nm	TEOA	50	-	[14] *Chem* **2019**, *5*, 1632-1647.
	sp^2c-COF_{ERDN}	>420 nm	TEOA	50	0.46 (420 nm)	[14] *Chem* **2019**, *5*, 1632-1647.
	g-$C_{40}N_3$-COF	>420 nm	TEOA	50	4.84 (420 nm)	[15] *Nat. Commun*. **2019**, *10*, 2467.
polymers	CP-CMP10	>420 nm	diethylamine	100	-	[17] *J. Am. Chem. Soc*. **2015**, *137*, 3265-3270.
	OB-POP-3	>420 nm	TEOA	50	2.0 (420 nm)	[18] *Adv. Funct. Mater*. **2017**, 27, 1703146.
	P7	>420 nm	MeOH/TEA	25	7.2 (420 nm)	[19] *Angew. Chem. Int. Ed.* **2016**, *55*, 1792-1796.
	B-BT-1.4	>420 nm	TEOA	50	4.01 (420 nm)	[20] *Angew. Chem. Int. Ed.* **2016**, *55*, 9202-9206.
	PTEPB	>420 nm	no	20	10.3 (420 nm)	[21] *Adv. Mater*. **2017,** *29*, 1702428.
	PyBT-2	>420 nm	TEOA	100	-	[22] *Appl. Catal. B: Environ.* **2018**, *228*, 1-9.
	P10	>420 nm	TEA	25	11.6 (420 nm)	[23a] *Nat. Commun*. **2018**, *9*, 4968
	P64	AM 1.5	TEA	25	20.7 (420 nm)	[23b] *J. Am. Chem. Soc*. **2019**, *141*, 9063-9071
	ter-CTF-0.7	>420 nm	TEOA	50	22.8 (420 nm)	[23c] *ACS Catal.* **2019**, *9*, 9438−9445
	FSO-FS	>420 nm	TEOA	50	6.8 (420 nm)	[23d] *Angew. Chem. Int. Ed.* **2019**, *58*, 10236 –10240.

Table S5. Inductively coupled plasma mass spectrometry (ICP-MS) determination of palladium (Pd) impurity in materials.

Materials	COF-alkene	COF-imide	COF-imine	TPAL-polymer	BPDA-polymer
Pd/ppm	0.53	0.14	0.06	0.10	0.52

References

[1] S. Jiang, J. Bacsa, X. F. Wu, J. T. A. Jones, R. Dawson, A. Trewin, D. J. Adams, A. I. Cooper, *Chem. Commun.* **2011**, *47*, 8919-8921.

[2] X. D. Zhuang, W. Zhao, F. Zhang, Y. Cao, F. Liu, S. Bi, X Feng, *Polym. Chem.* **2016**, *7*, 4176-4181.

[3] E. Q. Jin, M. Asada, Q. Xu, S. Dalapati, M. A. Addicoat, M. A. Brady, H. Xu, T. Nakamura, T. Heine, Q. H. Chen, D. L. Jiang, *Science* **2017**, *357*, 673-676.

[4] Q. R. Fang, Z. B. Zhuang, S. Gu, R. B. Kaspar, J. Zheng, J. H. Wang, S. L. Qiu, Y. S. Yan, *Nat. Commun*. **2014**, *5*, 4503.

[5] H. Xu, J. Gao, D. L. Jiang, *Nat. Chem.* **2015**, *7*, 905.

[6] A. Pecchia, A. Di. Carlo, *Rep. Prog. Phys*. **2004**, *67*, 1497-1561.

[7] A. Pecchia, G. Penazzi, L. Salvucci, A. D. Carlo, *New J. Phys*. **2008**, *10*, 065022.

[8] a) S. Q. Xu, G. Wang, B. P. Biswal, M. Addicoat, S. Paasch, W. B. Sheng, X. D. Zhuang, E. Brunner, T. Heine, R. Berger, X. L. Feng, *Angew. Chem. Int. Ed*. **2019**, *58*, 849-853. b) E. Q. Jin, J. Li, K. Y. Geng, Q. H. Jiang, H. Xu, Q. Xu, D. L. Jiang, *Nat. Commun*. **2018**, *9*, 4143. c) Y. J. Zhao, H. Liu, C. Y. Wu, Z. H. Zhang, Q. Y. Pan, F. Hu, R. M. Wang, P. W. Li, X. W. Huang, Z. B. Li, *Angew. Chem. Int. Ed*. **2019**, *58*, 5376-5381.

[9] T. Banerjee, F. Haase, G. Savasci, K. Gottschling, C. Ochsenfeld, B. V. Lotsch, *J. Am. Chem, Soc*. **2017**, *139*, 16228-16234.

[10] P. Pachfule, A. Acharjya, J. Roeser, T. Langenhahn, M. Schwarze, R. Schomacker, A. Thomas, J. Schmidt, *J. Am. Chem. Soc*. **2018**, *140*, 1423-1427.

[11] L. Stegbauer, K. Schwinghammer, B. V. Lotsch, *Chem. Sci*., **2014**, *5*, 2789-2793.

[12] V. S. Vyas, F. Haase, L. Stegbauer, G. Savasci, F. Podjask, C. Ochsenfeld, B. V. Lotsch, *Nat. Commun*. **2015**, *6*, 8508.

[13] X. Y. Wang, L. J. Chen, S. Y. Chong, M. A. Little, Y. Z. Wu, W. H. Zhu, R. Clowes, Y. Yan, M. A. Zwijnenburg, R. S. Sprick, A. I. Cooper, *Nat. Chem*. **2018**, *10*, 1180-1189.

[14] E, Q, Jin, Z. A. Lan, Q. H. Jiang, K. Y. Geng, G. S. Li, X. C. Wang, D. L. Jiang, *Chem* **2019**, *5*, 1632-1647.

[15] S. Bi, C. Yang, W. B. Zhang, J. S. Xu, L. M. Liu, D. Q. Wu, X. C. Wang, Y. Ham, Q. F. Liang, F. Zhang, *Nat. Commun*. **2019**, *10*, 2467.

[16] a) Q. Han, C. Hu, F. Zhao, Z. P. Zhang, N. Chen, L. T. Qu, *J. Mater. Chem. A*, **2015**, *3*, 4612–4619; b) Y. Chen, P. D. Tran, P. Boix, Y. Ren, S. Y. Chiam, Z. Li, K. W. Fu, L. F. Wong, J. Barber, *ACS nano*, **2015**, *9*, 3829-3836.

[17] R. S. Sprick, J. X. Jiang, B. Bonillo, S. J. Ren, T. Ratvjitvech, P. Guiglion, M. A. Zwijnenburg, D. J. Adams, A. I. Cooper, *J. Am. Chem. Soc.* **2015**, *137*, 3265-3270.

[18] S. Bi, Z. A. Lan, S. Paasch, W. B. Zhang, Y. F. He, C. Zhang, F. Liu, D. Q. Wu, X. D. Zhuang, E. Brunner, X. C. Wang, F. Zhang, *Adv. Funct. Mater*. **2017**, 27, 1703146.

[19] R. S. Sprick, B. Bonillo, R. Clowes, P. Guiglion, N. J. Brownbill, B. J. Slater, F. Blanc, M. A. Zwijnenburg, D. J. Adams, A. I. Cooper, *Angew. Chem. Int. Ed.* **2016**, *55*, 1792-1796.

[20] C. Yang, B. C. Ma, L. Z. Zhang, S. Lin, S. Ghasimi, K. Landfester, K. A. I. Zhang, X. C. Wang, *Angew. Chem. Int. Ed*. **2016**, *55*, 9202-9206.

[21] L. Wang, Y. Y. Wan, Y, J, Ding, S. K. Wu, Y. Zhang, X. L. Zhang, G. Q. Zhang, Y. J. Xiong, X. J. Wu, J. L. Yang, H. X. Xu, *Adv. Mater*. **2017**, *29*, 1702428.

[22] Y. F. Xu, N. Mao, C. Zhang, X. Wang, J. H. Zeng, Y. Chen, F. Wang, J. X. Jiang, *Appl. Catal. B: Environ*. **2018**, *228*, 1-9.

[23] a) M. Sachs, R. S. Sprick, D. Pearce, S. A. Hillman, A. Monti, A. A. Guilbert, N. J. Brownbill, S. Dimitrov, X. Y. Shi, F. Blanc, M. A. Zwijnenburg, J. Nelson, J. R. Durrant, A. I. Cooper, *Nat. Commun*. **2018**, *9*, 4968; b) Y. Bai, L. Wilbraham, B. J. Slater, M. A. Zwijnenburg, R. S. Sprick, A. I. Cooper, *J. Am. Chem. Soc*. **2019**, *141*, 9063-9071; c) L. P. Guo, Y. L. Niu, S. Razzaque, B. Tan, S. B. Jin, *ACS Catal*. **2019**, *9*, 9438−9445; d) Z. A. Lan, G. G. Zhang, X. Chen, Y. F. Zhang, K. A. Zhang, X. C. Wang, *Angew. Chem. Int. Ed.* **2019**, *58*, 10236 –10240.
